# Semi-Crystalline Polyoxymethylene-*co*-Polyoxyalkylene Multi-Block Telechels as Building Blocks for Polyurethane Applications

**DOI:** 10.3390/polym14050882

**Published:** 2022-02-23

**Authors:** Matthias Hoffmann, Matthias Hermesmann, Matthias Leven, Walter Leitner, Thomas Ernst Müller

**Affiliations:** 1 CAT Catalytic Center, RWTH Aachen University, Worringerweg 2, 52074 Aachen, Germany; matthias.hoffmann1@rwth-aachen.de (M.H.); matthias.leven@covestro.com (M.L.); leitner@itmc.rwth-aachen.de (W.L.); 2 Carbon Sources and Conversion, Ruhr-Universität Bochum, Universitätsstraße 150, 44801 Bochum, Germany; hermesmann@ls-csc.rub.de; 3 Covestro Deutschland AG, COV-CCO-PUR-R&D-EMEA-DRDII, B108, 51365 Leverkusen, Germany; 4 Max Planck Institute for Chemical Energy Conversion, Stiftstrasse 34–36, 45470 Mulheim an der Ruhr, Germany

**Keywords:** polymer building block, telechel, diol, polyol, copolymerization, oxymethylene moiety, polyurethane, NMR spectroscopy, kinetics, sustainability, carbon footprint

## Abstract

Hydroxy-terminated polyoxymethylene-*co*-polyoxyalkylene multi-block telechels were obtained by a new methodology that allows for the formal substituting of ether units in polyether polyols with oxymethylene moieties. An interesting feature is that, unlike carbonate groups in polycarbonate and polyethercarbonate polyols, homopolymer blocks of polyoxymethylene moieties can be formed. The regular nature of polyoxymethylene blocks imparts a certain crystallinity to the polymer that can give rise to new properties of polyurethanes derived from such telechels. The synthesis, reaction sequence and kinetics of the formation of oligomeric hydroxy-terminated multi-block telechel polyoxymethylene moieties are discussed in this paper and the preparation of a polyurethane material is demonstrated.

## 1. Introduction

**Scheme 1:**
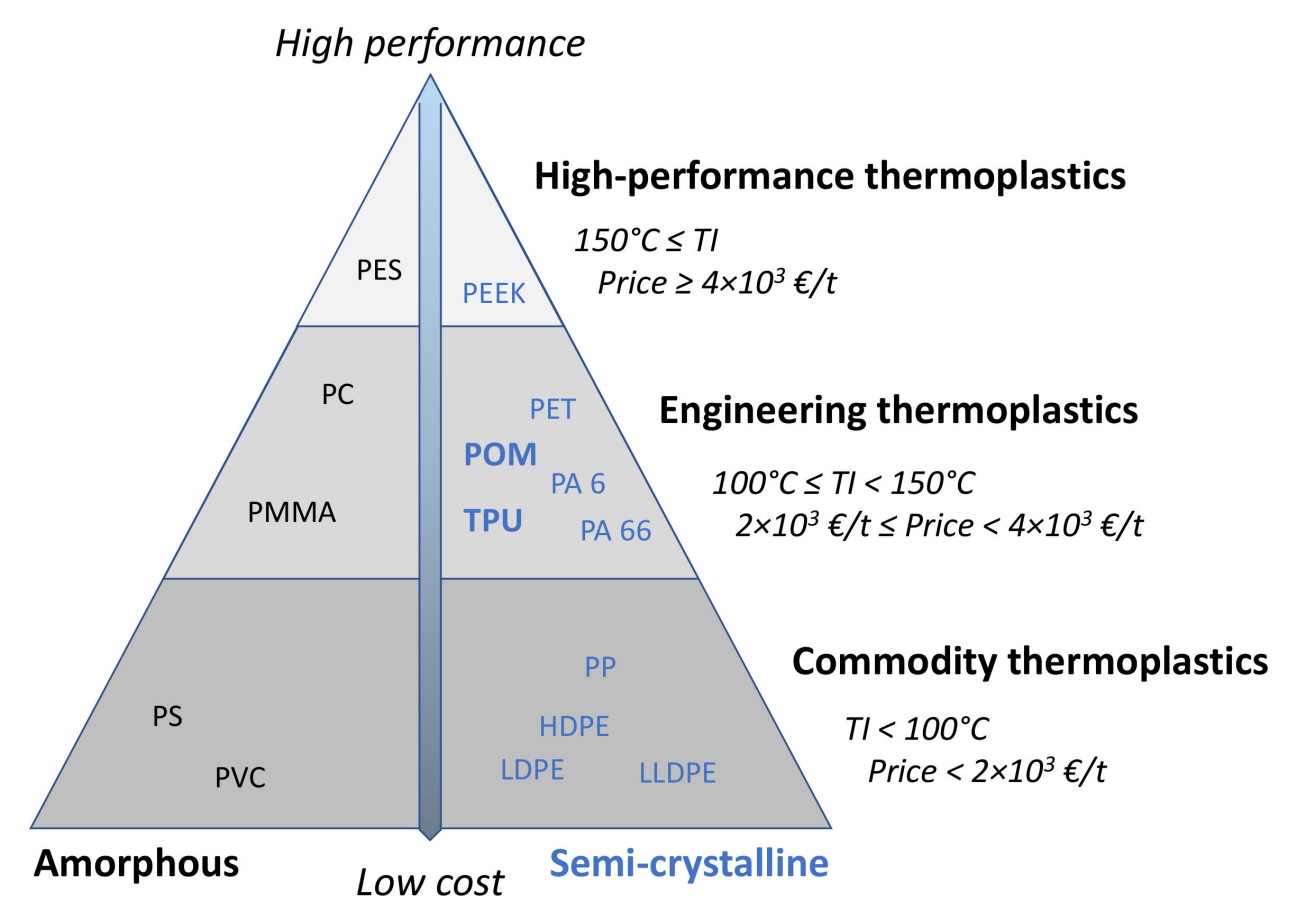
Classification of thermoplastic polyurethanes (TPU) and polyoxymethylene (POM) within the systematics of engineering thermoplastics [15]. TI, temperature index by Underwriter Laboratories. From top to bottom, *amorphous*: PES, polyethersulfone; PC, polycarbonate; PMMA, polymethylmethacrylate; PS, polystyrene; PVC, polyvinylchloride. *Semi-crystalline*: PEEK, polyetheretherketone; PET, polyethyleneterephthalate; PA 6, polyamide-6; PA 66, polyamine-66; PP, polypropylene; HDPE, high-density polyethylene; LDPE, low-density polyethylene; LLDPE, linear low-density polyethylene.

Engineering plastics [1] are produced at present mostly with monomers originating from petrochemical feedstock [2,3]. Aiming to make such materials more sustainable, intensive research is directed at replacing fossil-based monomers with monomers that can be derived from sustainable feedstock [4,5,6,7,8]. An important class of polymeric materials are high-performance polyurethanes (PU) [9,10,11,12,13,14]. The largest market share is for flexible and rigid PU foams, while thermoplastic polyurethanes (TPU) have a smaller market share. TPU are classified [15] as engineering thermoplastics [16] that are characterized by a higher performance compared to commodity thermoplastics and their capability for preserving structural integrity at higher operation temperatures above 100 °C (Scheme 1). Base polymers that allow for operation temperatures above 150 °C are typically referred to as high-performance thermoplastics. Properties relevant to engineering plastics comprise a high mechanical strength and good resistance to impact and chemicals. Improved performance is built on the chemical structure of the base polymer. The elaborate production route required to generate the base polymer and lower production volumes results in a higher price range. Depending on their propensity to form crystalline domains, engineering thermoplastics are grouped into amorphous and semi-crystalline polymers [15]. The closest representatives to the polymers investigated in this study (infra vide) are thermoplastic polyurethane (TPU) and polyoxymethylene (POM). While TPU form crystalline domains, POM is characterized by a strong propensity to crystallize. Typical representatives of amorphous engineering thermoplastics are polycarbonate (PC) and polymethylmethacrylate (PMMA). Semi-crystalline engineering thermoplastics include polyesters (PET) and polyamides (PA 6, PA 66). These polymers are applied extensively in the automotive, electronics, medical and other industrial sectors.

The sustainability of polyurethane (PU) materials that are produced mostly by reacting polyols with polyisocyanates [9,10,11,12,13,14] benefits from incorporating carbon dioxide as a co-monomer into the polyol [17,18,19]. As a chemical building block, carbon dioxide has a comparatively low but not zero global warming impact (GWI) [20]. By copolymerizing carbon dioxide with epoxides, such as propylene oxide and/or ethylene oxide, polyols ranging from polyethercarbonate polyols (–[(CH_2_CHRO)_m_–C(O)–O]_n_–; R = Me, H; m > 1) [21,22] to alternating polycarbonate polyols (–[CH_2_CHRO–C(O)–O]_n_–) [23,24,25,26,27,28,29] are readily obtained [13,30]. With an increasing fraction of carbonate groups, the viscosity of polyethercarbonate polyols also increases [31], making the processing of the building blocks in PU foam manufacture more arduous. As a compromise between low viscosity and the maximum carbonate content, polyethercarbonate polyols with an intermediate carbonate content of 20–30 mol-% and m ≈ 2 are often the preferred oligomeric building blocks in the production of polyurethane materials [13,32].

In search for alternative monomers for polyol manufacture [33,34,35,36,37,38,39] that, in principle, can be sourced from sustainable or renewable resources (Table 1), we became interested in hydroxy-terminated polyoxymethylene (POM)-based telechels as a potential substitute for polyethercarbonate polyols [40,41,42,43]. Unlike the carbonate groups in polyethercarbonate polyols, polyoxymethylene blocks can be present as homopolymer moieties [44,45]. This gives rise to a higher weight fraction of oxygen atoms that can be built into the polyols. This increases sustainability and leads to a decrease in the energy content of the polyols [42]. The incorporation of POM moieties in polyurethanes has been related to environmental benefits that can be traced back to indirect CO_2_ utilization [46]. Further, oxymethylene moieties are susceptible to cleavage by acids and certain enzymes, making polyurethanes derived from oxymethylene-containing polyols potentially biodegradable [47]. With regard to the production value chain, polyoxymethylene-containing polymer building blocks can be obtained from formaldehyde [44,45] that is produced on a large scale by the partial oxidation of methanol [48,49]. At this point it shall be noted that methanol can be obtained also from renewable resources [50,51] by CO_2_ hydrogenation [52,53,54,55], electrochemically [56,57,58] or fermentation with special micro-organisms [59,60,61]. The direct synthesis of oxymethylene units in linear and cyclic acetals from CO_2_ and H_2_ has also been described recently [62]. Like this, the forthcoming utilization of polyoxymethylene blocks for polymer manufacture can be traced back to sustainable resources [63]. For example, a paraformaldehyde-based approach to generate such polymer structures is currently investigated as part of the Kopernikus project “P2X” funded by the Ministry of Research and Education in Germany [64].

Overall, the production of polyurethanes is currently related to a relatively high global warming impact (GWI, Table 1). Both the main building blocks, polyisocyanates and polyol, contribute to the GWI of polyurethanes. Rigid foam polyurethanes are related to a GWI of 4.59 kg CO_2_-eq./kg (market mix). Flexible foam polyurethanes are related to GWIs of 3.67–3.28 and 3.04 kg CO_2_-eq./kg for polyurethanes derived from toluene di-isocyanate (TDI) and methylene diphenyl di-isocyanate (MDI), respectively. The polyol component contributes a GWI of 4.11–4.01 kg CO_2_-eq./kg (production mix). Polyether polyols that have a GWI of 3.91–3.03 kg CO_2_-eq./kg have a significant market share. The prevailing building blocks, ethylene oxide and propylene oxide, contribute 2.44–1.41 and 1.79 kg CO_2_-eq./kg, respectively. Thus, the upstream chains with the production of the chemical building blocks have to be addressed in order to lower the GWI of polyurethanes. Due to its lower GWI of 1.04–0.94 kg CO_2_-eq./kg compared to other monomers for polyol production, formaldehyde is of particular interest. Note that formaldehyde is produced mostly by the partial oxidation of methanol. Methanol produced in the conventional way by steam reforming contributes a GWI of 0.70–0.66 kg CO_2_-eq./kg, and that produced by biomass gasification contributes 0.69–0.67 kg CO_2_-eq./kg.

Due to their regular structure, polyoxymethylenes tend to give rise to high crystallinity, which impairs processing. The block length therefore needs to be controlled for POM-containing telechels to be used as polyurethane building blocks. The approach pursued in this paper was to use appropriate comonomers. Accordingly, we explored the use of short-chain glycols as comonomers, and a method was devised to synthesize hydroxy-terminated block-copolymer telechels with an OH functionality $\left(f\right)$ of two. Similar to high-molecular-weight polyoxymethylenes, such as Delrin [65], that are stabilized by copolymerizing trioxane with small amounts of a comonomer that gives rise to oxyethylene groups such as dioxolane [48] or ethylene oxide [48,66], we aimed to incorporate a certain fraction of oxyethylene groups that function as a zip-stopper, inhibiting the degradation of the polymer chains [48], and at the same time give rise to stable primary hydroxy end groups. Consequently, this study addresses the copolymerization of polyoxyalkylene diols (HO-POA-OH), with trioxane as the formaldehyde source and dioxolane as the comonomer. A kinetic analysis of the copolymerization reaction revealed insights into the elementary steps that control the block lengths in the polyoxymethylene-*co*-polyoxyalkylene multi-block telechels that are obtained. Last but not least, the applicability of such telechels as a building block for polyurethane manufacture is demonstrated.

## 2. Materials and Methods

### 2.1. Materials

Chemicals: chloroform-*d*^1^, 99.6% (Deutero GmbH, Kastellaun, Germany); dichlorobenzene, anhydrous, 99% (Sigma-Aldrich Chemie GmbH, Taufkirchen, Germany), secure seal, withdrawn under counter current argon flow; dichloromethane, anhydrous, ≥99.8% (Sigma-Aldrich), secure seal, withdrawn under counter current argon flow; dioxolane, anhydrous, 99.8% (Sigma-Aldrich), secure seal, withdrawn under counter current argon flow; *n*-pentane, technical, >98% (Haltermann Carless, Hamburg, Germany); sulfur trioxide DMF complex, 97+ % (ACROS Organics, Geel, Belgium); triethylamine, >98% (Fluka Analytical, Merck KGaA, Darmstadt, Germany); trioxane, ≥99% (Sigma-Aldrich). These were used as supplied if not stated otherwise. Polyoxyethylene diols **A**, **B**, and **C** were obtained with nominal weights of 1000, 400 and 200 g/mol, respectively, as PEG 1000, (Fluka, Honeywell Specialty Chemicals Seelze GmbH, Seelze, Germany), PEG 400, (Fluka), and PEG 200 for synthesis (Merck KGaA, Darmstadt, Germany); they were then dried in an oil pump vacuum prior to used. The number average molecular weights were determined with gel permeation chromatography, being 1076, 364 and 136 g/mol, respectively.

#### 2.1.1. Synthesis of Copolymer **1** with Diol **A**

Under inert conditions, a 3-neck flask was charged with 1,3,5-trioxane (40.62 g, 0.45 mol, 4.9 eq.) and dioxolane (26.03 g, 0.35 mol, 3.8 eq.). Diol **A** (1078 g/mol, nominal 1000 g/mol, 92.65 g, 0.093 mol, ≡1.0 eq.) and 1,2-dichlorobenzene (132 mL) were added. The mixture was then heated to 60 °C. After the required temperature was reached, the SO_3_·DMF complex (6.998 g, 0.046 mol, 0.5 eq., 0.25 eq. per hydroxy-group) was added. The reaction mixture was stirred for 24 h. During this time, aliquots of 1 mL volume were withdrawn, quenched by addition to an aqueous solution of triethylamine and analyzed by GPC chromatography and NMR spectroscopy. The reaction was stopped by adding triethylamine (2.530 g, 0.025 mol, 0.5 eq.) and deionized water (0.450 g, 0.025 mol, 0.5 eq.). The mixture was stirred for another 10 min. The mixture was then filtered through a paper filter. The filtrate was dissolved in dichloromethane (200 mL) and precipitated 3 times in 1 L of *n*-pentane. The supernatant was removed by decantation and the remaining grey gel-like substance was dissolved in dichloromethane. The mixture was filtered through celite. The volatiles were removed in a partial vacuum, and the residue was dried in a high vacuum. The residue was taken up in dichloromethane, filtered through silica gel and the product was precipitated with *n*-pentane. After drying under high vacuum, copolymer **1** was obtained as a white solid (93.62 g, 59% yield). Analytic data on copolymer **1** are provided in the Supplementary Materials Table S2.

#### 2.1.2. Synthesis of Copolymers **10**–**13** with Diol **A**

Under inert conditions, a 3-neck flask was charged with 1,3,5-trioxane (2.702 g–27.024 g, 0.03–0.3 mol, 1–10 eq.) and dioxolane (8.890 g, 0.12 mol, 4.0 eq.). Diol **A** (1078 g/mol, nominal 1000 g/mol, 30 g, 0.03 mol, ≡1.0 eq.) and 1,2-dichlorobenzene (44 mL) were added. The mixture was then heated to 60 °C. After the required temperature was reached, the SO_3_·DMF complex (2.297 g, 0.015 mol, 0.5 eq., 0.25 eq. per hydroxy-group) was added. The reaction mixture was stirred for 24 h. During this time, the viscosity of the reaction mixture increased and a highly viscous white suspension formed. The reaction was stopped by adding dichloromethane (200 mL), triethylamine (1.572 g, 0.015 mol, 0.5 eq.) and deionized water (0.270 g, 0.015 mol, 0.5 eq.). The mixture was stirred for another 10 min. The mixture was then filtered through a paper filter. The filtrate was dissolved in dichloromethane and precipitated 3 times in 1 L of *n*-pentane. The supernatant was decanted and the remaining grey gel-like substance was dissolved in dichloromethane. The mixture was filtered through celite. The volatiles were removed in a partial vacuum, and the residue was dried in a high vacuum. The residue was taken up in dichloromethane, filtered through silica gel and the product was precipitated with *n*-pentane. After drying under high vacuum, copolymers **10**–**13** were obtained as a greyish wax-like solid material in 57, 60, 69, and 66 wt-% yields, respectively. Analytic data on copolymers **10**–**13** are provided in the Supplementary Materials Table S2.

#### 2.1.3. Synthesis of Copolymers **6**–**9** with Diol **B**

Under inert conditions, a 3-neck flask was charged with 1,3,5-trioxane (4.504 g–45.040 g, 0.05–0.5 mol, 1–10 eq.) and dioxolane (14.816 g, 0.2 mol, 4.0 eq.). The starter diol B (364 g/mol, nominal 400 g/mol, 20 g, 0.05 mol, ≡1.0 eq.) and 1,2-dichlorobenzene (44 mL) were added. The mixture was then heated to 60 °C. After the required temperature was reached, the SO_3_·DMF complex (3.829 g, 0.025 mol, 0.5 eq., 0.25 eq. per hydroxy-group) was added. The reaction mixture was stirred for 24 h. During this time, the viscosity of the reaction mixture increased and a highly viscous white suspension formed. The reaction was stopped by adding triethylamine (2.530 g, 0.025 mol, 0.5 eq.) and deionized water (0.450 g, 0.025 mol, 0.5 eq.). The mixture was stirred for another 10 min. The mixture was then filtered through a paper filter. The filtrate was dissolved in dichloromethane and precipitated 3 times in 1 L of *n*-pentane. The supernatant was decanted and the remaining grey gel-like substance was dissolved in dichloromethane. The mixture was filtered through celite. The volatiles were removed in a partial vacuum, and the residue was dried in a high vacuum. The residue was taken up in dichloromethane, filtered through silica gel and the product was precipitated with *n*-pentane. After drying under high vacuum, copolymers **6**–**9** were obtained as a greyish wax-like solid material in 67, 59, 70, and 46 wt-% yields, respectively. Analytic data on copolymers **6**–**9** are provided in the Supplementary Materials Table S2.

#### 2.1.4. Synthesis of Copolymers **2**–**5** with Diol **C**

Under inert conditions, a 3-neck flask was charged with 1,3,5-trioxane (4.504 g–45.040 g, 0.05–0.5 mol, 1–10 eq.) and dioxolane (14.816 g, 0.2 mol, 4.0 eq.). Diol **C** (136 g/mol, nominal 200 g/mol, 10 g, 0.05 mol, ≡1.0 eq.) and 1,2-dichlorobenzene (44 mL) were added. The mixture was then heated to 60 °C. After the required temperature was reached, the SO_3_·DMF complex (3.829 g, 0.025 mol, 0.5 eq., 0.25 eq. per hydroxy group) was added. The reaction mixture was stirred for 24 h. During this time, the viscosity of the reaction mixture increased and a highly viscous white suspension formed. The reaction was stopped by adding triethylamine (2.530 g, 0.025 mol, 0.5 eq.) and deionized water (0.450 g, 0.025 mol, 0.5 eq.). The mixture was stirred for another 10 min. The mixture was then filtered through a paper filter. The filtrate was dissolved in dichloromethane and precipitated 3 times in 1 L of *n*-pentane. The supernatant was decanted and the remaining grey gel-like substance was dissolved in dichloromethane. The mixture was filtered through celite. The volatiles were removed in a partial vacuum, and the residue was dried in a high vacuum. The residue was taken up in dichloromethane, filtered through silica gel and the product was precipitated with *n*-pentane. After drying under high vacuum, copolymers **2**–**5** were obtained as a greyish wax-like solid material in 34, 27, 55, and 32 wt-% yields, respectively. Analytic data on copolymers **2**–**5** are provided in the Supplementary Materials Table S2.

#### 2.1.5. Synthesis of Polyurethane **14**

Under inert conditions, a 3-neck flask was charged with copolymer **1** (20.17 g, 0.0024 mol, 1 eq.) and 120 mL of dry dichlorobenzene. To this solution, 14.7 mg of DBTL (1 mol-%) was added and the resulting mixture was heated to 80 °C. Upon reaching the desired temperature, 0.77 eq. of toluene di-isocyanate (TDI) (0.33 g, 0.75 eq.) was added, followed by another addition of TDI after 1 h (0.22 g, 0.5 eq.) and again after 2 h (0.26 g, 0.6 eq.) (Figure S8). After 24 h, the heating was stopped. Upon reaching room temperature the reaction product was precipitated by the addition of *n*-pentane. The precipitate was dissolved in dichloromethane. The volatiles were removed in a partial vacuum. After drying under an oil pump vacuum, polyurethane **14** was obtained as a yellow solid (16.00 g, 76% yield, M_n_ (GPC) = 10.6 × 10^3^ g/mol).

### 2.2. Methods

#### 2.2.1. Nuclear Magnetic Resonance (NMR) Spectroscopy

The ^1^H and ^13^C NMR spectra were recorded on a Bruker AV400 spectrometer. The spectra were analyzed with the software TopSpin 3.2. Deuterated chloroform-d^1^ was used as solvent if not stated otherwise. The ^1^H NMR spectra were referenced to the chloroform solvent peak at 7.26 ppm. The ^13^C NMR spectra were referenced to the chloroform solvent peak at 77.16 ppm.

#### 2.2.2. Infra-Red (IR) Spectroscopy

An alpha IR tabletop spectrometer of the company Bruker with the software OPUS 7.0 was utilized to record the IR spectra.

#### 2.2.3. In Situ Infra-Red (IR) Spectroscopy

In situ IR spectra were recorded with an IR matrix FM TNo. 120200MX from Bruker and an IR probe equipped with a diamond window. The software OPUS 7.0 was used to record the spectra. The deconvolution of the recorded IR spectra was performed with the software PEAXACT V3 of S-PACT, Aachen, Germany [67]. To obtain concentrations of measured IR spectra, a concentration series of single components was measured. The recorded spectra were used to calibrate the concentrations of the components.

#### 2.2.4. Gel Permeation Chromatography (GPC)

The chromatograms were recorded on a purchased Agilent Technologies SECurity GPC. As detector, a refraction-index detector (RID) was used. Polystyrene references supplied by PSS were utilized to calibrate the system. Chloroform or THF were utilized as solvent and eluent (flow rate 1 mL/min). The molecular weight of the polymers was measured relative to polymer standards of known molecular weight. The software WinGPC version 8.10 by PSS Polymer Standard Service was used for recording and evaluating the gel permeation chromatograms. The data treatment is described in the Supplementary Materials Figures S1–S4.

#### 2.2.5. Differential Scanning Calorimetry (DSC)

The measurements were performed on a Mettler Toledo DSC1/500/1703 machine. The sample (5–15 mg) was placed in an aluminum crucible, and the difference in the heat flow between the sample crucible and a reference crucible was recorded by following the temperature program set as described in the main text. The data were analyzed with the STAR^e^ software version 11.00a.

#### 2.2.6. Thermogravimetric Analysis (TGA)

The measurements were performed on a Mettler Toledo TGA/DCC1 LF/1165 machine. The sample (20–40 mg) was placed in an aluminum oxide crucible, and the weight of a sample was recorded as the temperature rose. The data were analyzed with the STAR^e^ software version 11.00a.

### 2.3. Data Treatment and Kinetics

The data treatment and the kinetic model are described in detail in the Supplementary Materials. To determine the rate constants for the addition of trioxane and dioxolane to the growing chain end, the concentration profiles obtained with NMR were recalculated to a logarithmic plot (Figure S7) and the rate constants were determined as the slope of the linear functions.

## 3. Results and Discussion

**Scheme 2:**
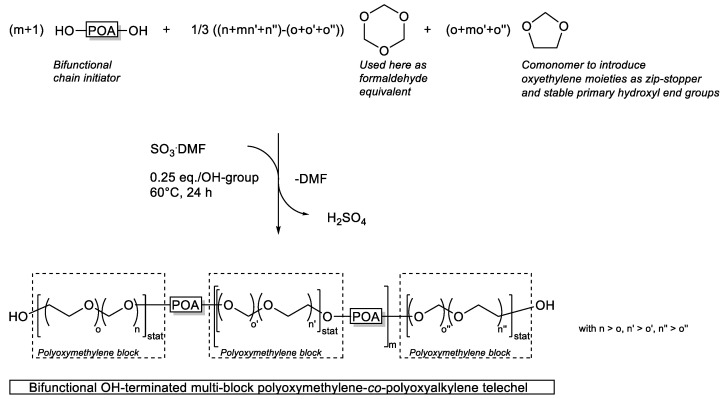
Sulfur trioxide-initiated formation of multi-block polyoxymethylene-*co*-polyoxyalkylene copolymers from polyoxyalkylene diols (HO-POA-OH), trioxane and dioxolane.

Multi-block polyoxymethylene-*co*-polyoxyalkylene polymers were obtained from short-chain polyoxyalkylene diols (HO-POA-OH) and trioxane. Through the use of dioxolane as a comonomer, the oxymethylene blocks were interrupted in a statistical manner by oxyethylene units. The copolymerization reaction (Scheme 2) was initiated with sulfur trioxide, which was employed as a dimethylformamide complex (SO_3_·DMF). The reaction of sulfur trioxide with a hydroxyl group of HO-POA-OH eliminates a hydroxide ion from the diol chain end, forming an oxiranium cation [68,69,70]. The oxiranium cation initiates the cationic ring opening polymerization of trioxane and dioxolane. The competing reaction of the oxiranium with a hydroxy group of another chain results in the condensation of two growing chains and terminates the chain growth reaction. This gives rise to the multi-block structure of the target polyoxymethylene-*co*-polyoxyalkylene telechels shown in Scheme 2.

### 3.1. Synthesis and Characterization of Copolymer **1**

Telechelic multi-block copolymer **1** was obtained by reacting polyoxyethylene (POE) diol **A** (M_n_ 1078 g/mol) with trioxane (4.9 eq.) and dioxolane (4.0 eq.) in the presence of SO_3_·DMF (0.25 eq. per hydroxy group). The number average molecular weight M_n_ of polyol **1** was determined with gel permeation chromatography (GPC) to be 8.2 × 10^3^ g/mol (see Supplementary Materials Figure S4). The molecular weight of the telechel is a result of the condensation reactions taking place. For the ratio of SO_3_·DMF to hydroxy groups of 0.25, the condensation of, on average, two chains to a dimeric oligomer was anticipated. A molecular weight of the telechel of 3.6 × 10^3^ g/mol was calculated for the full conversion of trioxane and dioxolane. The molecular weight measured by GPC was somewhat higher than the molecular weight anticipated. The observed higher molecular weight suggests that additional condensation reactions may have occurred.

**Figure 1:**
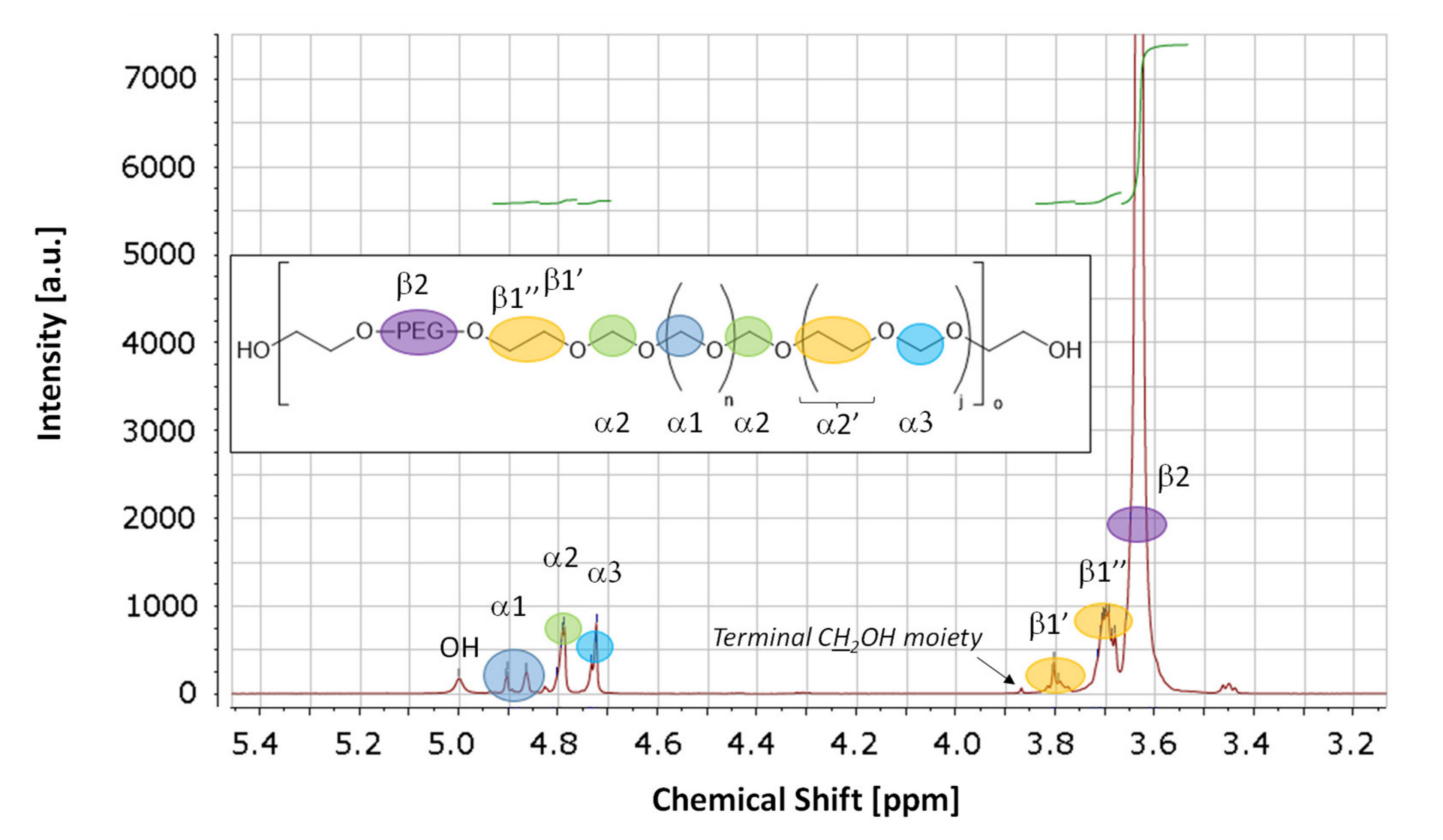
^1^H NMR spectrum of telechelic multi-block copolymer **1** and assignment of the signals.

**Figure 2:**
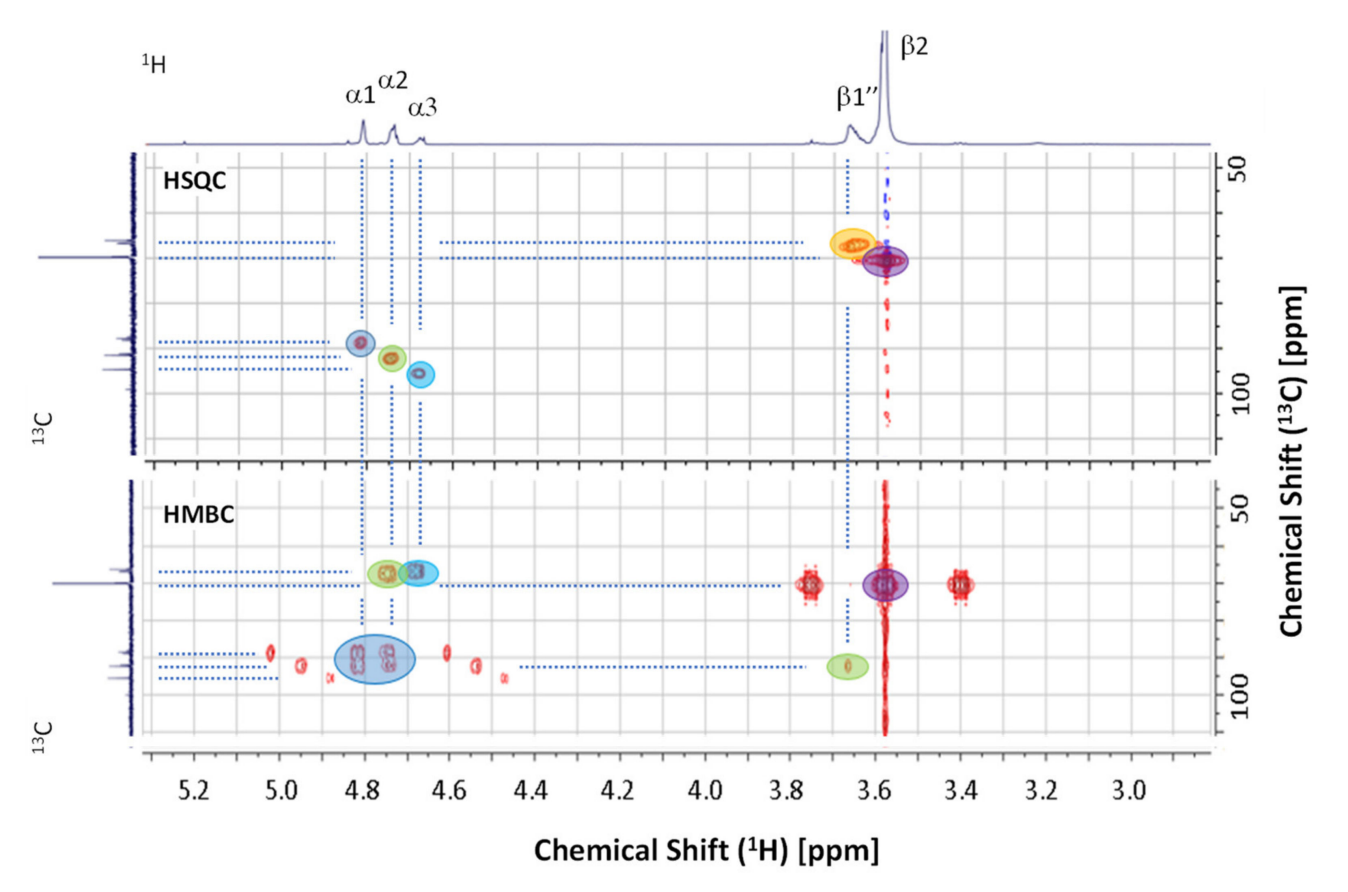
HSQC (**top**) and HMBC spectrum (**bottom**) of **1** revealing the ^1^H–^13^C coupling of protons to neighboring carbon atoms and the corresponding multi-bond coupling, respectively.

To resolve the molecular structure of copolymer **1**, the isolated sample was characterized in depth by NMR spectroscopy. In the ^1^H NMR spectra, distinct signals of oxymethylene and oxyethylene moieties can be differentiated (Figure 1). Multi-dimensional NMR spectroscopy was used to assign the signals (Table 2). The heteronuclear single quantum coherence (HSQC) spectrum of **1** (Figure 2, top) revealed the ^1^H→^13^C single bond coupling of the three groups of oxymethylene signals. The first group of signals, at a ^1^H chemical shift of 4.91–4.84 ppm (α1), related to the signals at 89.2–88.8 ppm in the ^13^C spectrum, was assigned to the inner oxymethylene moieties bound only to oxymethylene moieties. The second group of signals at 4.82–4.77 ppm (α2) in the ^1^H spectrum, related to the signals at 92.5–92.1 ppm in the ^13^C spectrum, was assigned to oxymethylene moieties connected to an oxyethylene and an oxymethylene moiety. The third group of signals at 4.75–4.71 ppm (α3) in the ^1^H spectrum, related to a signal at 95.6 ppm in the ^13^C spectrum, was assigned to isolated oxymethylene moieties with two neighboring oxyethylene moieties. In the same way, two characteristic groups of signals at 3.82–3.78 ppm and 3.74–3.66 ppm in the ^1^H spectrum, related to signals at 67.7–67.5 ppm and 67.0–66.8 ppm in the ^13^C spectrum, were assigned to the marginal oxyethylene moieties with a neighboring oxyethylene and a neighboring oxymethylene moiety. The pronounced signal at 3.66–3.57 ppm in the ^1^H spectrum was related to a signal at 70.7 ppm in the ^13^C spectrum and assigned to the oxyethylene moieties within the POE blocks.

The heteronuclear multiple bond coherence (HMBC) spectrum of **1** (Figure 2, bottom) revealed multi-bond couplings of the ^13^C NMR signals in the range of 67.7–66.8 ppm (marginal oxyethylene units) coupled to the ^1^H NMR signals at 3.66–3.57 ppm (oxyethylene groups within PEO blocks) and 4.82–4.77 and 4.75–4.71 ppm (oxymethylene groups). These findings confirm that oxyethylene units were connected to oxymethylene units. The ^1^H NMR signals at 4.75–4.71 ppm were observed to couple only to ^13^C NMR signals (67.7–67.5 and 67.0–66.8 ppm) assigned to oxyethylene units, indicating that these ^1^H NMR signals correspond to isolated oxymethylene units. The ^1^H NMR signals of oxymethylene units at 4.82–4.77 ppm were observed to couple to the ^13^C NMR signals of other oxymethylene units (92.5–92.1, 89.2–88.8 ppm) as well as oxyethylene units (67.7–66.8 ppm), indicating that these oxymethylene units were connected to oxyethylene and oxymethylene units. Furthermore, ^1^H NMR signals at 4.91–4.84 ppm were observed to couple only with ^13^C NMR signals assigned to oxymethylene units, but not to oxyethylene units. This suggests that the ^1^H NMR signals at 4.91–4.84 ppm correspond to inner oxymethylene units that are connected only to other oxymethylene units.

The composition of copolymer **1** was calculated based on the number average molecular weight (M_n_) and the relative intensities of the ^1^H NMR signals. The average content of polyoxymethylene (x_CH2O_) moieties was 20.3 mol-%. This corresponds to an average length of the polyoxymethylene blocks (n_CH2O_) of 7.5 units. The average number of repeating units per copolymer chain (o_avg_) was o_avg_ = 3.4. Provided all polyoxymethylene moieties were incorporated in sequence and condensation was the last step in the reaction sequence (vide infra), the upper limit for the length of the average polyoxymethylene block (n_CH2O_) was 9.4 units for the terminal groups and 18.8 units for the central polyoxymethylene block. The observed lower value for an n_CH2O_ of 7.5 units corresponds well to the introduction of additional oxyethylene units through the use of dioxolane as comonomer.

### 3.2. Reaction Sequence

**Figure 3:**
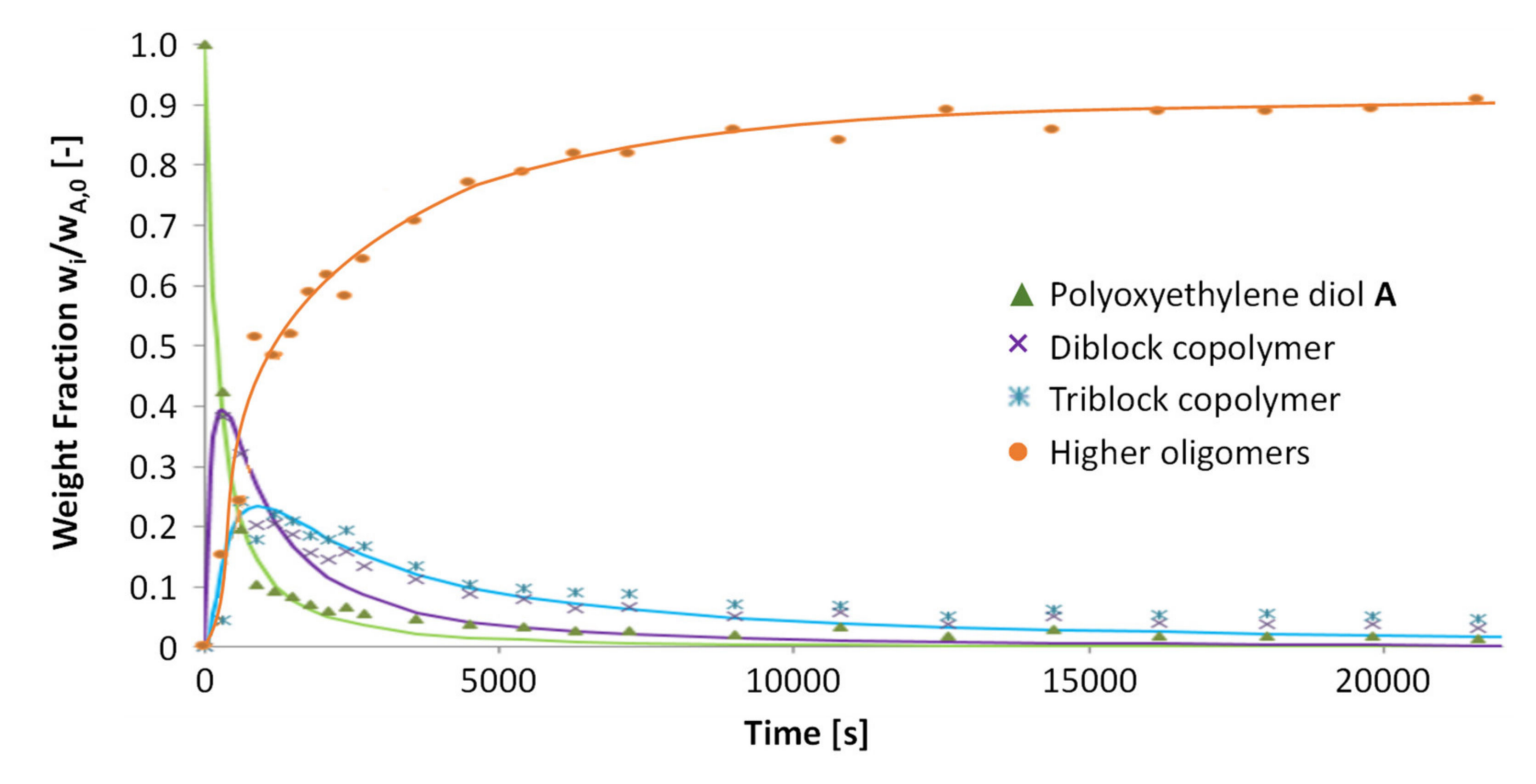
Profile of the species that occurred during the formation of copolymer **1**. The continuous lines provide a fit of the data with a second-order model according to differential equations D1 to D9 (see Supplementary Materials). For the higher oligomers, the continuous line gives a visual guide.

To obtain insight into the reaction sequence, aliquots were withdrawn recurrently during the copolymerization of trioxane and dioxolane in the presence of polyoxyethylene diol A to copolymer **1**. The aliquots were quenched through addition to an aqueous solution of triethylamine, and the composition of the mixtures was analyzed by ^1^H NMR spectroscopy. A plot of the concentrations as a function of time (Figure 3) revealed a rapid decrease in the weight fraction of diol **A**, while trioxane and dioxolane were consumed more slowly (see Supplementary Materials Figure S5). The consumption of all three reagents commenced without an induction period. Thus, the initiation of the polymerization reaction through the reaction of the hydroxy groups of **A** with SO_3_·DMF must have been very fast. Notably, a plot of the ratio of the trioxane to the dioxolane concentration against time showed a constant value (see Supplementary Materials Figure S6). This suggests that trioxane and dioxolane were consumed in parallel. From this, we infer that the oxymethylene and oxyethylene moieties that result from the ring opening of trioxane and dioxolane were built into the growing polymer chain in a statistical manner. Please note that the ring opening of trioxane gives rise to the formation of three oxymethylene moieties, and the ring opening of dioxolane to the formation of one oxymethylene and one oxyethylene moiety.

**Figure 4:**
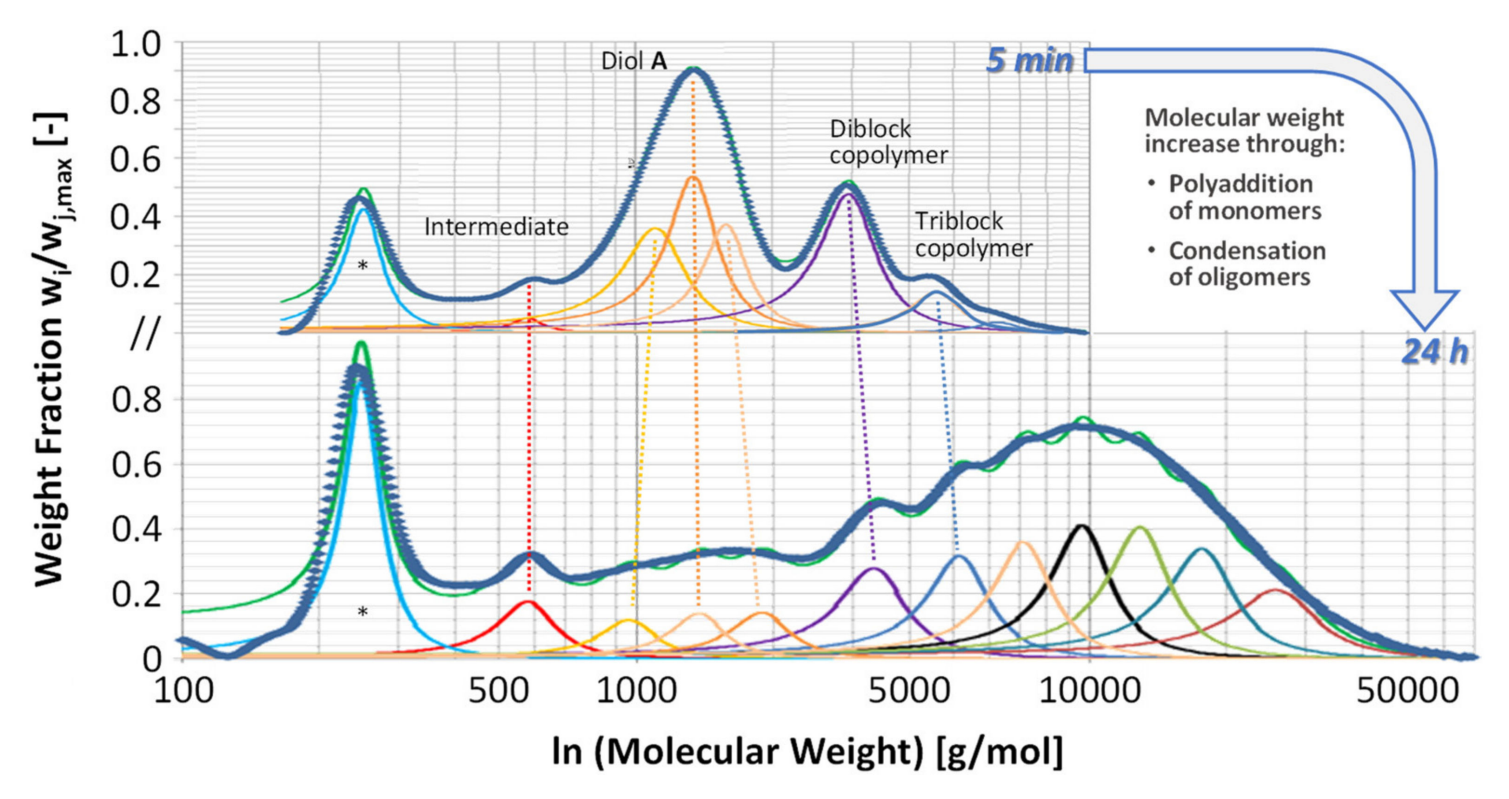
Molecular weight distribution (overlapping data points in blue color ♦) during the formation of copolymer **1** after 5 min reaction time (insert) and 24 h reaction time (bottom) and deconvolution of the distribution (continuous lines). The solvent peak is marked with an asterisk.

In parallel to the consumption of the monomers, oligomers with increasing molecular weights were formed. To obtain further insight, the molecular weight distribution was analyzed by gel permeation chromatography (GPC). An inspection of the chromatograms showed multi-modal molecular weight distributions that evolved steadily over time. For the sample withdrawn after 5 min (Figure 4), the molecular weight distribution showed four maxima at 585, 1300, 2830 and 4300 g/mol, respectively, and a high molecular weight tail above 5000 g/mol. These signals were assigned to a short-chain intermediate, a monomeric oligomer and diblock and triblock copolymers, respectively. The high-molecular-weight tail corresponds to oligomers with higher molecular weights.

Lorentz functions were used to deconvolute (see Supplementary Materials) the multi-modal molecular weight distributions. From the respective integral, the concentrations of the different oligomers were derived and the profiles are included in Figure 3. Concurrent to the consumption of **A**, the concentration of a dimeric species increased until a maximum was obtained after 5 min. The immediate onset of the formation of the dimer suggests that the dimeric species is a primary product. After 5 min, the concentration of the dimeric species decreased. Concurrently, the concentration of a trimeric species increased until a maximum was reached after 10 min. The S-shaped curve of the initial profile of the trimer concentration is clearly indicative of the trimer being a consecutive product. The trimer is most likely formed by condensation of the dimeric species with diol **A**. Subsequently, the concentration of the trimer decreased. Over longer reaction times, the concentration of higher oligomers increased. After 6 h, most of the copolymer chains (≥90 weight-%) had been converted to higher oligomers.

**Scheme 3:**
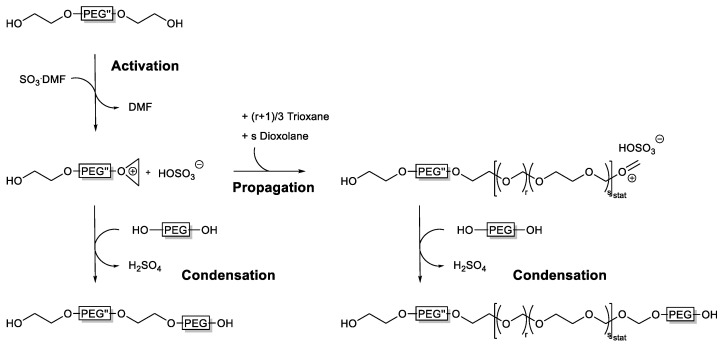
Likely chain propagation and condensation reaction pathways in the sulfur trioxide-initiated formation of multi-block polyoxymethylene-*co*-polyoxyethylene copolymers. POA″ designates the parent polyoxyalkylene diol (HO-POA-OH) except for two oxyethylene moieties.

The concentration profiles are in agreement with the proposed polycondensation pathway. Hence, the following steps were considered for the formation of multi-block copolymers (Scheme 3): The reaction was initiated by the reaction of the terminal hydroxy groups of the short-chain polyoxyethylene diol with SO3·DMF (see also Scheme 2). The elimination of hydroxide results in the formation of a terminal oxiranium hydrogen sulfate. The cationic charge of the oxiranium cation was resonance stabilized by delocalization over three atoms. Two options for chain propagation seem likely: (i) The oxiranium cation reacts with one of the oxygen centers of trioxane. Opening of the trioxane ring leads to the elongation of the chain by three oxymethylene moieties and the formation of a terminal oxonium cation [71]. (ii) The opening of the dioxane ring leads to the elongation of the chain by one oxyethylene moiety and, analogously, the formation of a terminal oxonium cation. In either case, the oxonium cation can open the ring of the next trioxane or dioxolane molecule in the same way as the oxiranium cation, thereby allowing for chain propagation. The condensation of the oxiranium or the oxonium cation with a terminal hydroxy group of another chain under the release of a proton terminates chain growth. Thereby, two oligomer chains are connected to one another.

The molecular weights of the multi-block POM-POE copolymers obtained in this study were determined by the relative rates of propagation and condensation. In the absence of condensation, the full conversion of trioxane and dioxolane would lead to an increase in molecular weight from 1.1 × 10^3^ for the parent diol **A** to 1.8 × 10^3^ g/mol. Clearly, the formation of oligomers with higher molecular weights must have been a consequence of condensation reactions. The extent to which condensation reactions occur primarily depends on the ratio of the initiator SO_3_·DMF to terminal hydroxy groups of the polyoxyethylene diol. Each SO_3_·DMF complex ought to abstract one water molecule, thereby triggering one condensation reaction. The ratio of 0.25 used in the experiments would then lead to the dimerization of polyoxyethylene chains. Evidently, other reactions must have been present, which led to additional condensation reactions. The precipitation of plain polyoxymethylene chains (“paraformaldehyde”) removes water from the reaction mixture. One molecule of water is withdrawn from the reaction mixture for each plain polyoxymethylene chain that precipitates. Oligomers have a high propensity to precipitate when more than eight consecutive oxymethylene moieties are present in the molecule (vide infra). The formation of such chains is more pronounced with a high content of trioxane in the reaction mixture. Other side reactions might be caused by the sulfuric acid released in the condensation reaction. Sulfuric acid may be able to abstract a water molecule [72] from a terminal hydroxy group in the same way as the SO_3_·DMF complex [73]. This may lead to additional condensation reactions. Furthermore, oxymethylene chains may be split by sulfuric acid into a hydroxy-terminated and a cationic chain end [74]. Even though this reaction does not lead to the build-up of additional molecular weight, it would allow the insertion of a trioxane or dioxolane monomer in the middle of a copolymer chain.

### 3.3. Kinetic Model

**Scheme 4:**
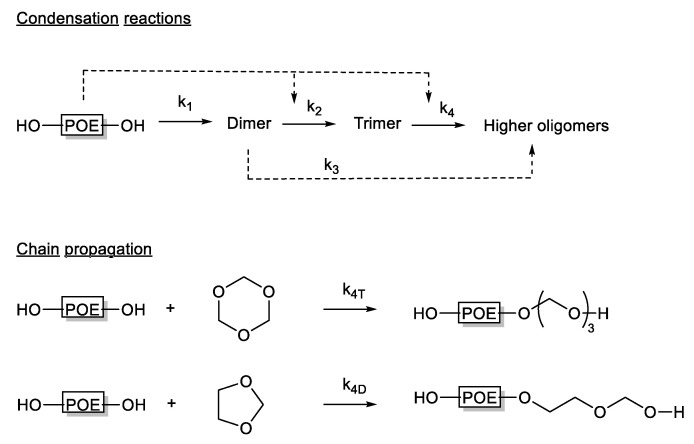
Reactions considered in the kinetic model for the formation of multi-block copolymer **1** from diol **A**, trioxane, and dioxolane.

The GPC profiles were described with a kinetic model (Scheme 4) that corresponds to a set of nine differential equations (D1–D9; see Supplementary Materials). The rate constants were determined by numerically fitting the kinetic model to the experimental data, which resulted in a good agreement of experimental and fitted data. An initial rate of r_ini_ = 1.4 × 10^−3^ (mol/L)·s^−1^ was determined for the consumption of **A**, which corresponds to a rate constant of k_1_ = 5.0 × 10^−3^ (mol/L)^−1^·s^−1^ for the condensation reactions involving **A**. The rate constant (k_2_ = 5.1 × 10^−3^ (mol/L)^−1^·s^−1^) for the condensation reactions of the dimer was determined to be in the same order of magnitude, while the rate constant for the condensation reactions (k_3_ = 2.5 × 10^−3^ (mol/L)^−1^·s^−1^) of the trimer and higher oligomers was found to be lower. This indicates that the rate constant decreases with an increasing length of copolymer chains once a critical length is obtained. The respective rate constants for the propagation reaction with trioxane (k_4T_ = −2.1 × 10^−5^ s^−1^) and dioxolane (k_4D_ = −5.4 × 10^−5^ s^−1^) were found to be two orders of magnitude lower.

### 3.4. Variation of Copolymer Composition

**Scheme 5:**
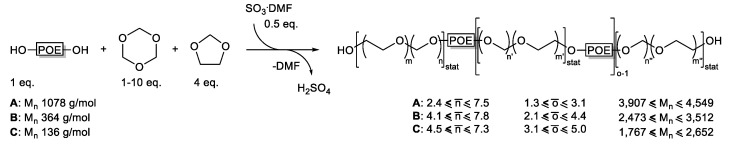
Sulfur trioxide-initiated formation of multi-block polyoxymethylene-*co*-polyoxyethylene copolymers from polyoxyethylene diols, trioxane, and dioxolane.

To explore the influence of the length of the short-chain polyoxyalkylene diol on the composition of polyoxymethylene-*co*-polyoxyethylene multi-block copolymers, polyoxyethylene diols **A**, **B**, and **C** with molecular weights of 1078, 364 and 136 g/mol, respectively, were employed (Scheme 5).

With a decreasing length of the polyoxyethylene diol, the number average molecular weight M_n_ of the copolymers decreased (Table 3). Thus, with 1.0–1.1 equivalents of trioxane, the number average molecular weight decreased from 3.9 × 10^3^ to 2.5 × 10^3^ and 1.8 × 10^3^ g/mol for copolymers **10**, **6**, and **2**. With 9.8–10.1 equivalents of trioxane, the number average molecular weight decreased from 4.5 × 10^3^ to 3.5 × 10^3^ and 2.7 × 10^3^ g/mol for copolymers **13**, **9**, and **5**, respectively. The fraction of oxymethylene moieties x_CH2O_ incorporated into the copolymer increased from 3.5 to 13.5 and 19.7 mol-% for copolymers **10**, **6**, and **2**. Likewise, the fraction of oxymethylene x_CH2O_ increased from 10.2 to 36.8 and 49.0 mol-% for copolymers **13**, **9**, and **5**, respectively. Clearly, the fraction of oxymethylene moieties x_CH2O_ increased as the parent polyoxyethylene diol shortened. In contrast, the average length of the oxymethylene block $\bar{n}$_CH2O_ showed less variation. It increased from 2.4 to 4.1 and 4.5 mol-% for copolymers **10**, **6**, and **2**, respectively. The average length of the oxymethylene block $\bar{n}$_CH2O_ varied around 7.5, 7.8, and 7.3 mol-% for copolymers **13**, **9**, and **5**, respectively. In this regard, the length of the parent polyoxyethylene diol had little influence. The average length of the oxymethylene blocks is consistent with the statistical incorporation of dioxolane and the presence of isolated oxyethylene moieties in the copolymer. The average number of repeating units per copolymer chain $\bar{o}$_avg_ increased from 1.3 to 2.1 and 2.0 mol-% for copolymers **10**, **6**, and **2**, respectively. Likewise, the average number of repeating units $\bar{o}$_avg_ increased from 3.1 to 4.4 and 5.0 mol-% for copolymers **13**, **9**, and **5**, respectively. This reflects an increased propensity towards condensation reactions the shorter the parent polyoxyethylene diol is.

The trioxane fraction had a strong effect on the number average molecular weight of the copolymers and was therefore varied systematically for each of the three parent polyoxyethylene diols. The number average molecular weights of copolymers **10** to **12** that were obtained from polyoxyethylene diol **A** increased from 3.9 × 10^3^ to 4.0 × 10^3^ and 4.6 × 10^3^ g/mol with increasing fraction of trioxane (1.1, 3.0, and 4.8 equivalents, respectively). With a further increase in the trioxane fraction to 9.8 equivalents, the number average molecular weight decreased somewhat to 4.5 × 10^3^ g/mol (copolymer **13**). This trend was even more pronounced when considering the fraction of oxymethylene units in the copolymers. Upon raising the trioxane fraction in the reaction mixture, the fraction of oxymethylene units in the copolymers x_CH2O_ increased from 3.5 to 13.5 and 20.7 mol-% for copolymers **10** to **12**, respectively, and then decreased to 10.2 mol-% for copolymer **13**. It is noteworthy that the copolymer yield was slightly lower for copolymer **13** than for the other copolymers due to the precipitation of plain polyoxymethylene chains. This is consistent with a pronounced propensity for the homo-polymerization of trioxane in the presence of higher trioxane concentrations. For copolymers **6** to **9**, which were obtained from polyoxyethylene diol **B**, the number average molecular weight increased from 2.5 × 10^3^ to 3.3 × 10^3^, 3.3 × 10^3^ and 3.5 × 10^3^ g/mol with an increasing fraction of trioxane (1.0, 3.1, 5.0, and 10.1 equivalents, respectively). The similar weights of copolymers **7** and **8** should be noted here. The fraction of oxymethylene units in the copolymers x_CH2O_ increased steadily with the trioxane fraction in the reaction mixture from 13.5 to 34.7, 36.5 and 36.8 mol-% for copolymers **6** to **9**, respectively. For copolymers **2** to **5**, which were obtained from polyoxyethylene diol **A**, the number average molecular weight increased steadily from 1.8 × 10^3^ to 2.2 × 10^3^, 2.4 × 10^3^ and 2.7 × 10^3^ g/mol with an increasing fraction of trioxane (1.0, 3.0, 4.9 and 10.1 equivalents, respectively). The fraction of oxymethylene units in the copolymers x_CH2O_ increased with the trioxane fraction in the reaction mixture from 19.7 to 45.6 and 52.0 mol-% for copolymers **6** to **8**, respectively. For copolymer **9,** there was a pronounced decrease in the fraction of oxymethylene units. Please note that the choice of the polyoxyethylene diol determines the length of the polyoxyethylene blocks copolymers as the diol chain remains intact under our reaction conditions. Consequently, the length of the polyoxyethylene block was set by the length of the polyoxyethylene diol, while the length of the polyoxymethylene block increased with an increasing ratio of trioxane to polyoxyethylene diol in the reaction mixture.

**Figure 5:**
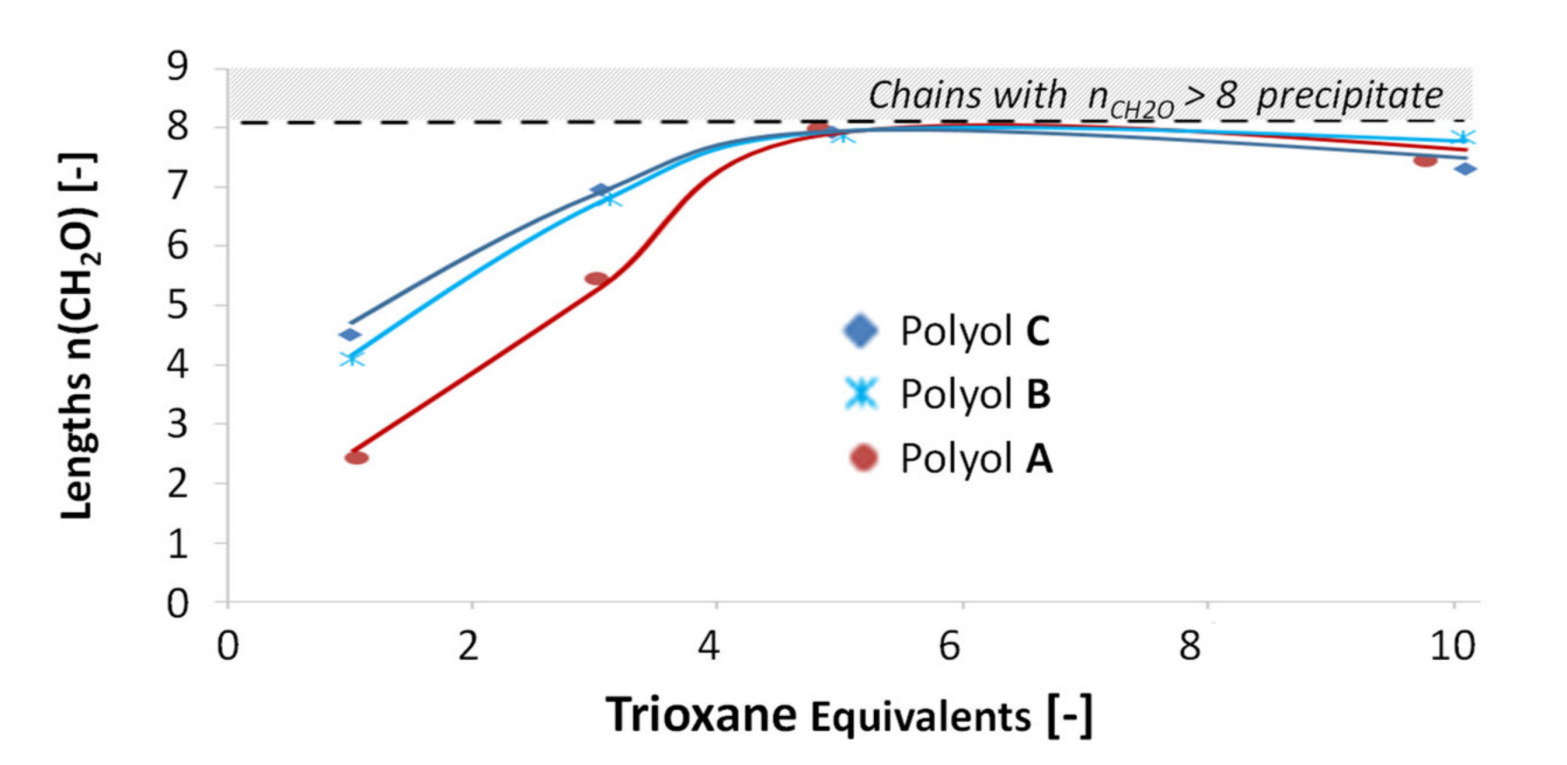
Influence of trioxane equivalents on the average length of the oxymethylene blocks in the polyoxymethylene-*co*-polyoxyethylene multi-block copolymers.

To obtain further insights, the average length of the oxymethylene blocks n_CH2O_ in the copolymers was plotted against the equivalents of the trioxane employed (Figure 5). Regardless of the choice of the diol, the average length of oxymethylene blocks increased with increasing trioxane equivalents to maximum values of 7.9, 7.9, and 8.0 at 5 eq. trioxane (copolymers **12**, **8**, and **4**, respectively). With a further increase in the trioxane equivalents, the average length of the oxymethylene blocks decreased to 7.5, 7.8, and 7.3 at 10 equivalents (copolymers **5**, **9** and **13**, respectively). At these high concentrations of trioxane, a higher proportion of an insoluble fraction was precipitated from the reaction mixture. Apparently, copolymer chains comprising oxymethylene blocks of more than eight repeating units tend to precipitate from the reaction mixture under our reaction conditions.

**Figure 6:**
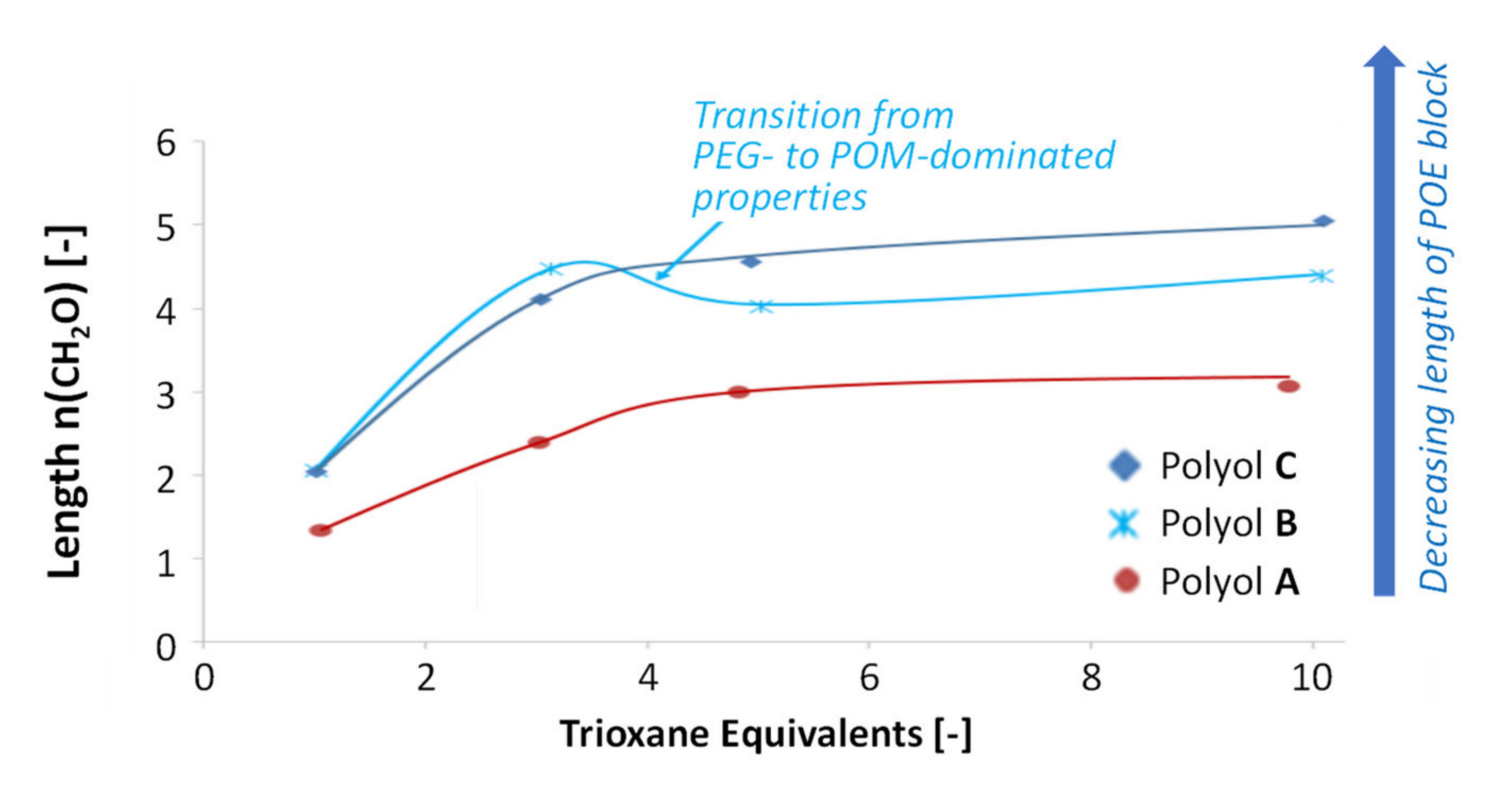
Influence of trioxane equivalents on the number of repeating blocks (o_avg_) for the poly-oxymethylene-*co*-polyoxyethylene multi-block copolymers.

In addition, the average number of repeating units per copolymer chain was plotted against the trioxane equivalents (Figure 6). For copolymers based on polyoxyethylene diol **A**, the average number of repeating units $\bar{o}$_avg_ increased with increasing trioxane equivalents from 1.3 to 2.4, 3.1 and 3.1 units for copolymers **10** to **13**, respectively. For copolymers based on polyoxyethylene diol **B**, the average number of repeating units $\bar{o}$_avg_ increased with increasing trioxane equivalents from 2.1 for copolymer **6** to level out at 4.5, 4.0 and 4.4 units for copolymers **7** to **9**, respectively. For copolymers based on polyoxyethylene diol **C**, the average amount of repeating units $\bar{o}$_avg_ increased with increasing trioxane equivalents from 2.0 for copolymer **2** to 4.1 for copolymer **3**, which was less pronounced at 4.6 and 5.0 units for copolymers **4** and **5**, respectively. Thus, with a higher ratio of trioxane equivalents, the number of repeating units per copolymer chain progressively exceeded the anticipated value of two. This finding is unexpected as the condensation of oligomer chains ought to depend only on the ratio of SO_3_·DMF to hydroxy groups. Clearly, there is a higher propensity for condensation reactions when more trioxane is present in the reaction mixture.

### 3.5. Mechanism of Multi-Block Copolymer Formation

**Scheme 6:**
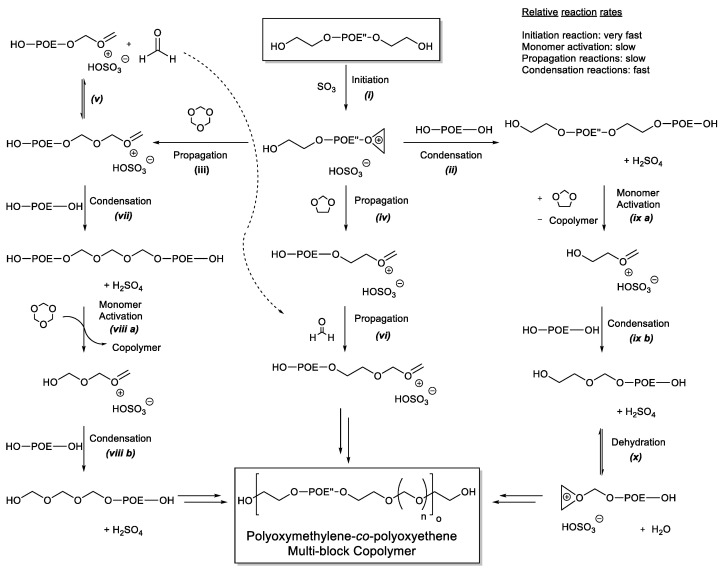
Reaction network proposed for the formation of polyoxymethylene-*co*-polyoxyethylene multi-block copolymers. Reactions (***v***) to (***x***) occur in a statistical manner. The alternative of multi-block POM-POE copolymer chains to react in condensation reactions (***vii***), (***viii b***) and (***ix b***) was omitted for clarity. Likewise, the reactions with further monomers forming growing homopolymer chains were omitted. The parent polyoxyethylene diol is abbreviated as HO-POE-OH, except for one oxyethylene moiety abbreviated as POE′, and except for two oxyethylene moiety abbreviated as POE″.

The proposed reaction network for the formation of polyoxymethylene-*co*-polyoxyethylene multi-block copolymers is exemplified in Scheme 6. The copolymerization is initiated by the reaction of a terminal hydroxy end group of the polyoxyethylene diol with SO_3_·DMF to form an ion pair comprising a terminal oxiranium cation and a hydrogen sulfate anion (***i***). This initial step is significantly faster than the subsequent propagation and condensation steps. The oxiranium cation can react with the hydroxy group of a polyoxyethylene diol (***ii***), the ether oxygen atom of trioxane (***iii***) or the ether oxygen of dioxolane (***iv***). The condensation of activated chain ends having hydroxy groups occurs quickly, thereby leading to the release of sulfuric acid (***ii***). The reaction leads to the connecting of two oligomer chains. The oxiranium cation reacts more slowly with trioxane or dioxolane in the propagation step (***iii*** and ***iv***). The addition of the monomers to the oxiranium cation that precedes the ring opening of the monomer is reversible (not shown). The ring is then opened and a terminal oxonium ion is formed. Once the propagation is underway, reactions (***v***) to (***x***) occur in a statistical manner. Terminal oxonium cations are prone to depolymerization [75], thereby releasing monomeric formaldehyde, whereby a steady-state concentration of monomeric formaldehyde is established in the reaction mixture (ceiling concentration, ***v***). As the concentration of monomeric formaldehyde increases, the oxonium cations react more frequently with monomeric formaldehyde in chain propagation reactions (***vi***). Similar to oxiranium cations, oxonium-terminated chains also react with the hydroxy groups of the parent polyoxyethylene diol or copolymer chains formed previously under the release of sulfuric acid (***vii***).

As the concentration of sulfuric acid increases over the course of the reaction, additional reactions become more likely. The protonation of trioxane and dioxolane by sulfuric acid induces the ring opening of the heterocycles (***viii a*** and ***ix a***). The subsequent condensation with hydroxy end groups leads to the elongation of the copolymer chain (***viii b*** and ***ix b***). Sulfuric acid may also react with hydroxy end groups, thereby dehydrating polymer chains to form new terminal oxiranium groups (***x***). Inversely released water molecules react with cationic chain ends, forming hydroxy end groups under the release of sulfuric acid. Several factors influence the propensity for dehydration and hydration reactions. The more sulfuric acid is released, the more likely dehydration reactions become. Growing polyoxymethylene chains become insoluble once a certain number of consecutive oxymethylene units is exceeded, thereby removing water from the reaction mixture. This leads to an increase in the ratio between cationic chain ends and hydroxy end groups, hence leading to an increase in the number of repeating units o_avg_.

**Scheme 7:**
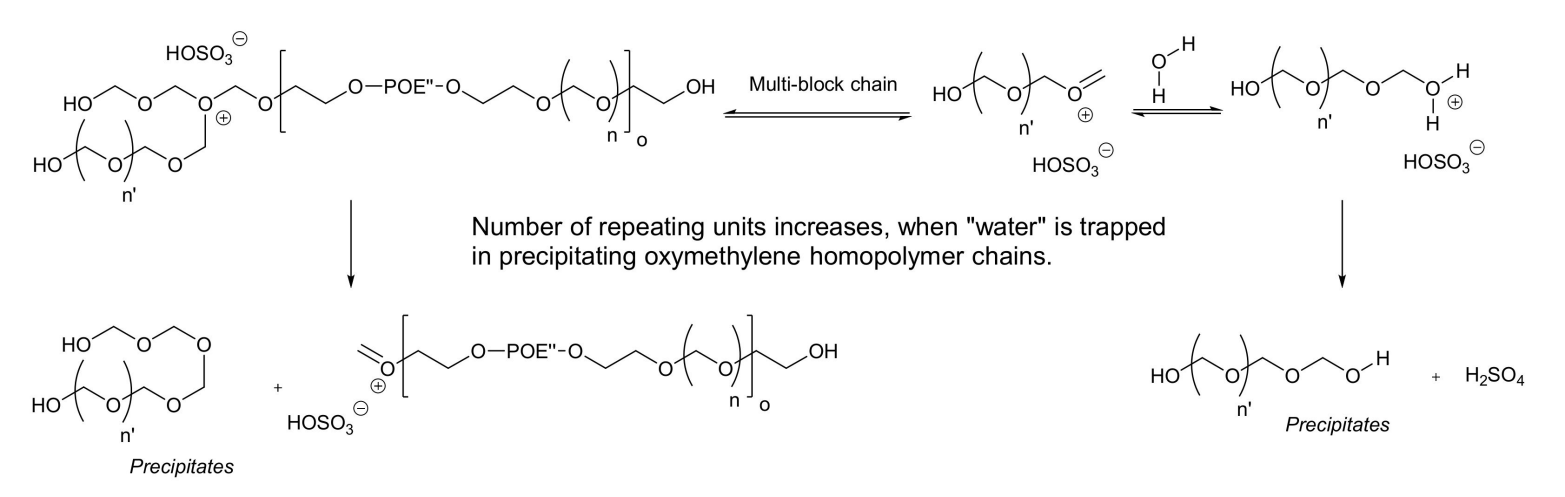
Likely steps in the formation of polyoxymethylene homopolymers. POE″ designates the parent polyoxyethylene diol (HO-POE-OH) except for two oxyethylene moieties.

Activated monomers and chain ends can also react with polyoxymethylene units by transacetalization [76]. This is exemplified for the reaction between an activated polyoxymethylene homopolymer chain and a multi-block copolymer chain, as shown in Scheme 7. In this case as well, plain polyoxymethylene chains have a strong tendency to precipitate, thereby removing water from the reaction mixture and leading to an increase in the average number of repeating units per copolymer chain $\bar{o}$_avg_.

### 3.6. Polyurethane Formation

**Figure 7:**
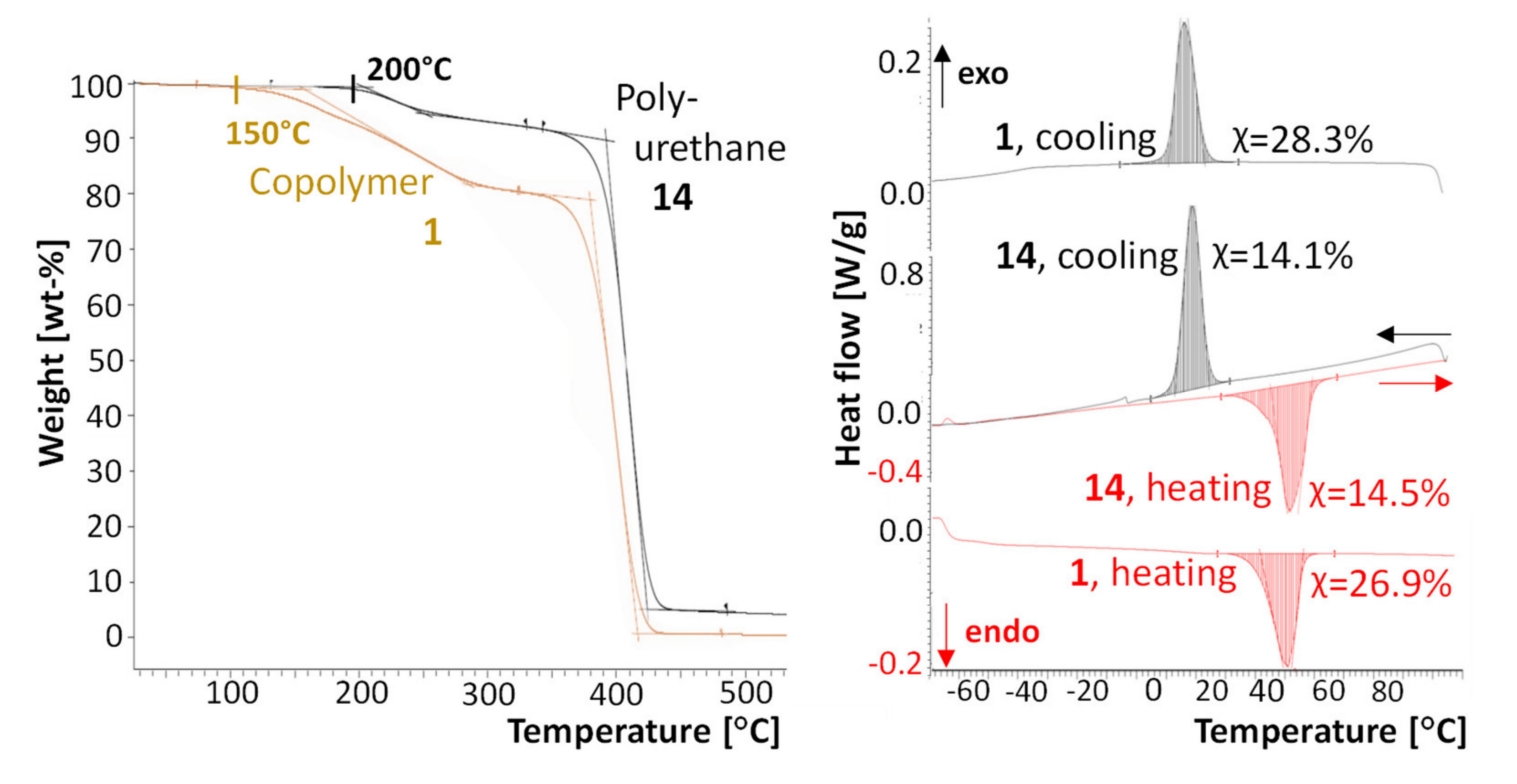
TGA measurement (**left**) and DSC traces (**right**) of copolymer **1** and polyurethane **14**. Samples were heated/cooled at 10 K/min.

For polyurethane synthesis, hydroxy-terminated polyoxymethylene-*co*-polyoxyethylene multi-block copolymer **1** was chain extended with toluene di-isocyanate in the presence of DBTL as catalyst. Polyurethane **14** was then precipitated from *n*-pentane. The characterization by ^1^H-NMR spectroscopy revealed that polyurethane **14** was composed of oxymethylene, oxyethylene, and polyurethane moieties. The number average molecular weight M_n_ of polyurethane **14** was determined by gel permeation chromatography (GPC) to be 10.6 × 10^3^ g/mol. The thermal stability was evaluated by considering thermogravimetry (TGA, Figure 7, left). The increase in the onset of thermal decomposition from 110 °C for the parent copolymer **1** to 200 °C for polyurethane **14** is consistent with successful chain elongation. While the parent copolymer **1** decomposed in two steps at 249 °C and 401 °C, with weight losses of 17% and 78%, respectively, polyurethane **14** decomposed at higher temperatures. For **14**, two steps at 229 °C and 415 °C led to weight losses of 8% and 85%, respectively. The first decomposition step was assigned to the polyoxymethylene moieties; the second decomposition step was assigned to the polyoxyethylene moieties. The increased stability of polyurethane **14** relative to **1** shows that chain elongation through the introduction of polyurethane units stabilizes oxymethylene moieties.

Last but not least, the phase transitions of copolymer **1** and polyurethane **14** were characterized by differential scanning calorimetry (DSC, Figure 7, right). Upon cooling, the parent copolymer **1** showed an exothermic signal between 29.47 and −12.26 °C. Upon heating, an endothermic signal was observed between 18.64 and 58.68 °C. Upon cooling, polyurethane **14** showed an exothermic signal between 25.1 and −2.7 °C. Upon heating, an endothermic signal was observed between 21.7 and 62.3 °C. The shape of the DSC traces suggests that, upon cooling, the copolymer solidifies by forming crystalline domains. Upon heating, these domains melt at a somewhat higher temperature. In comparison with a sample of neat polyoxymethylene, a degree of crystallinity of χ = 28% was determined for copolymer **1**. A significantly lower degree of crystallinity of χ = 14% was determined for polyurethane **14**. It is noteworthy that the width of the exothermal crystallization range decreased, while the range of the endothermal melting range increased slightly, when comparing copolymer **1** with the chain-elongated polyurethane **14**. Moreover, endothermal and exothermal signals shifted to higher temperatures when comparing the signals of copolymer **1** with polyurethane **14**. This suggests that the degree of crystallinity decreased, most likely due to the inhibition of oxymethylene alignment of the polymer chains prompted by the interaction of the urethane groups.

## 4. Conclusions

Multi-block polyoxymethylene-*co*-polyoxyethylene copolymers were obtained through the copolymerization of polyoxyethylene diols with trioxane and dioxolane. Such copolymers constitute a novel class of polyols that are suitable as sustainable building block for polyurethanes. The proposed production chain emanates from methanol obtained from sustainable sources. Methanol is used for producing formaldehyde which, in turn, is used for making trioxane and dioxolane. The copolymers produced from these monomers can have a high sustainable content that originates from the ability of oxymethylene moieties to form homopolymers. Of note, the maximum length of the polyoxymethylene blocks is limited to eight to avoid the precipitation of an insoluble fraction. Polyoxyethylene diols with molecular weights in the range of 136 to 1078 g/mol are shown to constitute suitable spacers to skirt the propensity of polyoxymethylene blocks to crystallize.

Due to the high propensity of oxymethylene homopolymers to form insoluble solids, polyoxymethylene diols have so far not been introduced in polyurethane chemistry. The methodology described in this study now provides access to suitable hydroxy-terminated block copolymers. Accordingly, the multi-block copolymer diols can be used for making polyurethanes. This was demonstrated by the chain elongation of a hydroxy-terminated copolymer with toluene di-isocyanate. The resulting copolymer was thermally stable up to temperatures of 200 °C. Neither copolymers nor polyurethane showed a glass transition. Instead, the polyurethane showed a pronounced tendency to crystallize. This makes semi-crystalline oligomeric building blocks an interesting target to replace 1,4-butanediol in PU rigid foam applications that are characterized by a certain crystallinity. Beyond polymer building blocks, another interesting application field of short-chain block copolymers would be as fuel additives.

## 5. Patents

The production of polyoxymethylene block copolymers is described by Gürtler, C., Müller, T. E., Leitner, W. and Leven, M. method for production of polyoxymethylene block copolymers, European Patent Application, EP 3287476 A1, priority date 28 February 2018.

The production of polyurethanes from polyoxymethylene-containing prepolymers is described by Gürtler, C., Müller, T. E., Bizzarri, C., Leven, M., Leitner, W. and Winkelhaus, D. Method for production of polyoxymethylene-containing prepolymer and method for production of polyurethane, European Patent Application, EP 3498743 A1, priority date 19 June 2019.

A method for the production of polyurethane polymers with crystallinity ≥9% used for manufacturing thermoplastics is described by Gürtler, C., Müller, T. E., Bizzarri, C., Leven, M. and Leitner, W. Method for the production of polyurethane polymers, European Patent Application, EP 3498746 A1, priority date 19 June 2019.

## Figures and Tables

**Table 1:** Global warming impact of selected monomers for polyol manufacture formed through different processes.

Level	Compound	GWI [kg CO_2_-eq./kg]	Source ^1^
Poly- urethane	Rigid foam	4.59	g-mp-EU
	Flexible foam, TDI-based	3.67–3.28	g-mp-EU
	Flexible foam, MDI-based	3.04	g-mp-EU
Iso- cyanate	TDI	4.76–3.22	g-mp-EU
	MDI	3.82–2.85	g-mp-EU
Polyol	Production mix	4.11–4.01	e-mp/up-EU/RoW
	Polyether polyol	3.91–3.03	g-mp-EU
Co- monomer	Ethylene oxide	2.44–1.41	g/e-mp/up-EU/RoW
	Propylene oxide, oxirane process	1.79	g-mp-D
	Formaldehyde	1.04–0.94	g/e-mp/up-EU/RoW
Pre- cursor	Methanol, steam reforming	0.70–0.66	e-mp/up-RoW
	Methanol, biomass gasification	0.69–0.67	e-mp/up-RoW

^1^ Details on the source of the data are provided in the Supplementary Materials Table S1. The source is encoded in the table as follows: ecoinvent v3.8 (e) or GaBi 2021.2 (g), market process (mp) or unit process (up) for Germany (D), Europe (EU), Global (G) or the Rest of World (RoW).

**Table 2:** Assignment of the signals in the ^1^H and ^13^C{^1^H} NMR spectra of telechelic multi-block copolymer **1** [13].

Signal	Assignment	δ(^1^H)	δ(^13^C)
α1	Inner oxymethylene moieties bound only to oxymethylene moieties	4.91–4.84	89.2–88.8
α2	Oxymethylene moieties connected to an oxyethylene and oxymethylene moiety	4.82–4.77	92.5–92.1
α3	Isolated oxymethylene moieties with neighboring oxyethylene moieties	4.75–4.71	95.6
β1′, β1″	Marginal oxyethylene moieties with a neighboring oxymethylene moieties	3.82–3.78, 3.74–3.66	67.7–67.5, 67.0–66.8
β2	Oxyethylene moieties within POE block	3.66–3.57	70.7

**Table 3:** Composition of polyoxymethylene-*co*-polyoxyethylene multi-block copolymers obtained by reacting polyoxyethylene diols **A**, **B** and **C** with trioxane and dioxolane in the presence of SO_3_·DMF.

Co- Polymer	Diol ^a^ [g/mol]	Eq._Tx_^b^ [-]	M_n_^c^ [g/mol]	x_CH2O_^d^ [mol-%]	$\bar{n}$_CH2O_^e^ [-]	$\bar{o}$_avg_^f^ [-]
**10**	**A**	1.1	3.9 × 10^3^	3.5	2.4	1.3
**11**	**A**	3.0	4.0 × 10^3^	13.5	5.5	2.4
**12**	**A**	4.8	4.6 × 10^3^	20.7	8.0	3.0
**13**	**A**	9.8	4.5 × 10^3^	10.2	7.5	3.1
**6**	**B**	1.0	2.5 × 10^3^	13.5	4.1	2.1
**7**	**B**	3.1	3.3 × 10^3^	34.7	6.8	4.5
**8**	**B**	5.0	3.3 × 10^3^	36.5	7.9	4.0
**9**	**B**	10.1	3.5 × 10^3^	36.8	7.8	4.4
**2**	**C**	1.0	1.8 × 10^3^	19.7	4.5	2.0
**3**	**C**	3.0	2.2 × 10^3^	45.6	7.0	4.1
**4**	**C**	4.9	2.4 × 10^3^	52.0	7.9	4.6
**5**	**C**	10.1	2.7 × 10^3^	49.0	7.3	5.0

^a^ The molecular weights of polyoxyethylene diols **A**, **B**, and **C** were 1078, 364 and 136 g/mol, respectively; ^b^ equivalents of trioxane (Tx); ^c^ number average molecular weight; ^d^ molar fraction of oxymethylene units incorporated; ^e^ average length of oxymethylene blocks; ^f^ average number of repeating units per copolymer chain.

## Data Availability

Data is contained within the article or the Supplementary Materials.

## Supplementary Materials

The following supporting information can be downloaded at: https://www.mdpi.com/article/10.3390/polym14050882/s1, detailed information on materials and methods, data treatment, analytic and kinetic data; supplementary analytic data on copolymers **1**–**13**; Figure S1: Relevant range of the elugram of copolymer **1**; Figure S2: Molecular weight distribution of copolymer **1**; Figure S3: Normalized molecular weight distribution of copolymer **1** measured by GPC chromatography in chloroform; Figure S4: Normalized molecular weight distribution of copolymer **1** measured by GPC chromatography in chloroform; Figure S5: Time–concentration profile of trioxane and dioxolane during the formation of copolymer **1**; Figure S6: Ratio of the concentrations of trioxane and dioxolane during the formation of copolymer **1**; Figure S7: Linearized time–concentration profile of the trioxane and dioxolane consumption during the formation of copolymer **1**; Figure S8: Isocyanate region of the time-resolved in situ IR spectra during the pulse-wise addition of TDI to copolymer **14**; Table S1: Global warming impact of polyurethane and polyurethane building blocks. Table S2: Multi-block POM-PEG copolymers synthesized from polyoxyethylene diols, trioxane, and dioxolane.

## Abbreviations

a.u.	arbitrary units
D	Germany
DMF	Dimethylformamide
DSC	Differential scanning calorimetry
e	ecoinvent v3.8
EU	Europe
eq.	equivalents
f	OH-functionality
g	GaBi 2021.2
G	Global
GPC	Gel permeation chromatography
HDPE	High density polyethylene
HMBC	Heteronuclear multiple bond coherence
HSQC	Heteronuclear single quantum coherence
LDPE	Low-density polyethylene
LLDPE	Linear low-density polyethylene
Me	Methyl
mp	market process
NMR	Nuclear magnetic resonance
PA	Polyamide
PC	Polycarbonate
PEEK	Polyetheretherketone
PEG	Polyethylene glycol
PES	Polyethersulfone
PET	Polyethyleneterephthalate
PMMA	Polymethylmethacrylate
POA	Polyoxyalkylene
POA′	Polyoxyalkylene except for a terminal oxyethylene group
POA″	Polyoxyalkylene except for two terminal oxyethylene groups
POE	Polyoxyethylene
POE′	Polyoxyalkylene except for a terminal oxyethylene group
POE″	Polyoxyalkylene except for two terminal oxyethylene groups
POM	Polyoxymethylene
PU	Polyurethane
PP	Polypropylene
PS	Polystyrene
PVC	Polyvinylchloride
R	Substituent
RoW	Rest of World
TGA	Thermal gravimetric analysis
TI	Capability by temperature index by Underwriter Laboratories
TPU	Thermoplastic polyurethanes
up	unit process
