# Supplementary material for: Semi-Crystalline Polyoxymethylene-co-Polyoxyalkylene Multi-Block Telechels as Building Blocks for Polyurethane Applications
Source: Polymers (Basel). 2022 Feb 23;14(5):882. doi: 10.3390/polym14050882 (PMC8912848; doi:10.3390/polym14050882)
Supplement: Supplementary file 1 [file polymers-14-00882-s001.zip › polymers-1572542-supplementary.pdf]

SUPPLEMENTARY INFORMATION

Semi-crystalline Polyoxymethylene-*co*-Polyoxyalkylene Multi-block Telechels as Building Blocks for Polyurethane Applications

Matthias Hoffmann, Matthias Hermesmann, Matthias Leven, Walter Leitner and Thomas E. Müller*

Global warming impact

Table S1 Global warming impact of polyurethane and polyurethane building blocks.

			Global Warming Impact [kg CO_2-eq./kg]							
			ecoinvent v3.8						GaBi 2021.2	
			Europe		Global		Rest of World		Europe	Germany
	Chemical compound	Production process	Market process	Unit process	Market process	Unit process	Market process	Unit process	Market process	Market process
Co-monomer	Ethylene oxide	Direct oxidation of ethylene	1,91	1,91			2,44	2,44	1,41	
		Oxidation of ethylene with air							1,52	1,56
		Production mix							2,03	
	Propylene oxide	From propene and chlorine (chlorohydrin process)	4,19	4,19			4,99	4,99		
		Chlorohydrin process with Ca(OH)2								4,53
		Chlorohydrin process with cell liquor							2,84	3,19
		From isobutane (Oxirane process)								1,79
	**Formaldehyd**	Oxidation of methanol (Formox process)	0,96	0,94			1,04	0,99		
		Dehydrogenation of methanol							1,01	1,02
	Succinic acid	Hydrogenation of maleic acid			3,28	3,23				
	Maleic anhydride	Catalytic oxidation of benzene		4,10	3,02			4,31		
		Direct oxidation of n-butane		2,20				2,59		
Pre-cursor	Methanol	Steam reforming of natural gas			0,70	0,66				
		From biomass gasification					0,69	0,67		
Polyol	Diethylene glycol	Hydrolysis of ethylene oxide								1,12
		Production mix							1,89	
	Triethylene glycol	Production mix							2,00	
	Polyol	Production mix	4,03	4,01			4,11	4,03		
	Polyether polyol	Production mix							3,91	
	Polyether polyol 5000 g/mol	Addition of propylene oxide and ethylene oxide							3,25	
	Long-chain polyether polyols, MW>1000g/mol	Polymerization of propylene oxide							3,03	
	Short-chain polyether polyols, MW<1000g/mol	Polymerization of propylene oxide							3,24	
Iso-cyanate	Methylene diphenyl diisocyanate (MDI)	Phosgenation of methylenedianiline	5,32	5,30			7,31	7,23	2,85	3,82
	Toluene diisocyanate (TDI)	Phosgenation of toluenediamine	6,92	6,92			6,93	6,93	3,22	4,76
Poly-urethane	Polyurethane, flexible foam	MDI-based, no flame retardant							3,04	
		TDI-based, no flame retardant, high density							3,31	
		TDI-based, no flame retardant, low density							3,28	
		TDI-based, with flame retardant, production mix							3,67	
	Polyurethane, rigid foam	Production mix							4,59	

* Polyether polyol 5000 g/mol, France

Analytical methods

Gel Permeation Chromatography (GPC)

The number average molecular weight (M_n) of copolymers **1** to **13** was determined gel permeation chromatography (GPC) in chloroform relative to polystyrene standard. The raw elugram of copolymer **1** showed a broad signal ranging from an elution volume of 14 mL chloroform until an elution volume of 22.5 mL solvent that had passed over the column. A sharp signal was observed after an elution volume of 24 mL (Fig. S1). In a first step of the analysis, the baseline was corrected, and the range for determining the molecular weight was chosen.

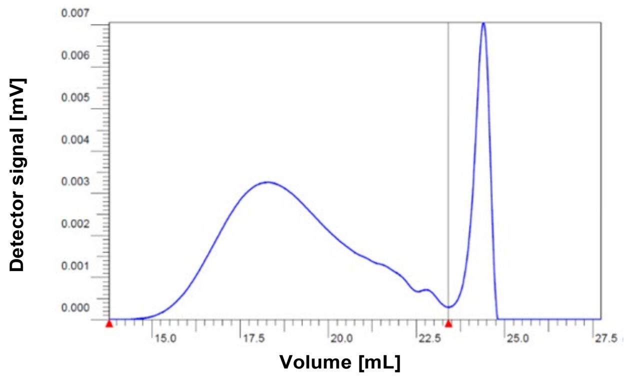

Figure S1 Relevant range of the elugram of copolymer **1**.

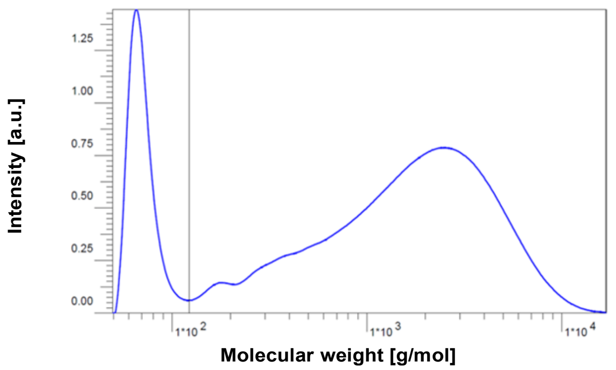

Figure S2 Molecular weight distribution of copolymer **1**.

The elution volume was recalculated to the molecular weight as shown in Figure S2. The signal observed below 100 g/mol is attributed to the presence of solvent molecules trapped inside of the polymer.
The range with a molecular weight above 900 g/mol was recalculated to the molecular weight of the corresponding copolymer fraction by referencing to polymer standards (polyoxyethylene diols) of known molecular weight (Figure S3). For copolymer **1,** a broad molecular weight distribution (PDI = 2.80) with a number average molecular weight (M_n) of 8,223 g/mol was found. Two low intensity signals were observed at molecular weights of below 5,000 g/mol.

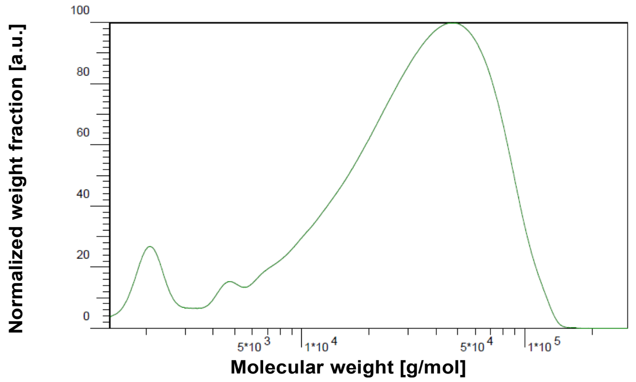

Figure S3 Normalised molecular weight distribution of copolymer **1** measured by GPC chromatography in chloroform (M_n = 8,223 g/mol, PDI = 2.80).

Data treatment

The concentrations of trioxane and dioxolane were derived according to the general procedure described below. The conversion factors (χ_i) were calculated according to Equation 1 whereby the term $c_{0,i}$ denotes the initial concentration of component *i*, and the term $A_{0,i}$ denotes the integral of component *i*.

$$\chi_i = \frac{c_{0,i}}{A_{0,i}} \qquad \text{Eq. 1}$$

The substrate concentrations were calculated according to Equation 2 with $c_{t,i}$: Concentration of component *i* after a time *t*; and $A_{t,i}$: Integral recorded for component *i* after a time *t*.

$$c_{t,i} = \chi_i \, A_{t,i} \qquad \text{Eq. 2}$$

The molar ratio (x_i) of inner and outer oxyethylene units as well as mono-, inner and outer oxymethylene units in the copolymer was determined from the ^{1}H NMR spectrum and was calculated according to equation 3 with x_i: Molar fraction of the moiety in mol-%; and v_i: Relative quantity of the moiety.

$$x_i = \frac{v_i}{v_{oCH2O}+v_{iCH2O}+v_{monoCH2O}+v_{oEO}+v_{iEO}} \times 100 \qquad \text{Eq. 3}$$

The relative quantity of the moieties was determined according to Equation 4:

$$v_i = {}^{A_i}/_{p_i} \qquad \text{Eq. 4}$$

Inserting Equation 4 into Equation 3 yields Equation 5 with A_i: Sum of integrals of the moiety; p_i: Number of hydrogen atoms in the moiety; *i*: Moiety; o_{CH2O}: Outer oxymethylene units; i_{CH2O}: Inner oxymethylene units; monoCH_2O: Mono-oxymethylene units; CH_2O: Oxymethylene moiety; o_{EO}: Outer oxyethylene units; i_{EO}: Inner oxyethylene units; and EO: oxyethylene.

$$x_i = \frac{{}^{A_i}/_{p_i}}{{}^{A_{oCH2O}}/_{p_{CH2O}}+{}^{A_{iCH2O}}/_{p_{CH2O}}+{}^{A_{monoCH2O}}/_{p_{CH2O}}+{}^{A_{oEO}}/_{p_{EO}}+{}^{A_{iEO}}/_{p_{EO}}} \times 100 \qquad \text{Eq.5}$$

The weight ratio (w_i) of inner and outer oxyethylene units as well as mono-, inner and outer oxymethylene units in the copolymer was determined according to Equation 6 with w_i: weight fraction of moiety *i* in wt.-%; and M_i: Molecular weight of moiety *i*.

$$w_i = \frac{v_i \times M_i}{v_{oCH2O}\times M_{oCH2O}+v_{iCH2O}\times M_{iCH2O}+v_{monoCH2O}\times M_{monoCH2O}+v_{oEO}\times M_{oEO}+v_{iEO}\times M_{iEO}} \times 100 \qquad \text{Eq. 6}$$

The unit amount (a_i) of inner and outer oxyethylene units as well as mono-, inner and outer oxymethylene units in the copolymer was calculated according to Equation 7 with a_i: amount of the units; and M_n: number average molecular weight of the copolymer.

$$a_i = \frac{w_i/100 \times M_n}{M_i} \qquad \text{Eq. 7}$$

It was assumed that the mono-oxymethylene units were formed mostly from dioxolane, so that one outer oxyethylene unit per mono-oxymethylene was introduced by the monomer. Moreover, these introduced oxyethylene units were assumed to be mostly connected to two oxymethylene blocks. Accordingly, the amount of outer oxyethylene units (a_{oiEO}) connected was calculated according to Equation 8:

$$a_{oiEO} = a_{oEO} - a_{monoCH2O} \qquad \text{Eq. 8}$$

The average length of the oxymethylene blocks (n_{CH2O}) of segments of more than one repeating unit was determined according to Equation 9:

$$n_{CH2O} = \frac{a_{iCH2O}+a_{oCH2O}}{a_{oiEO}} \qquad \text{Eq. 9}$$

The average length of the oxyethylene blocks (n_{EO}) of segments of more than one repeating unit was determined according to Equation 10:

$$n_{EO} = \frac{a_{iEO}+a_{oiEO}}{a_{oiEO}} \quad \text{Eq. 10}$$

The average amount of blocks (o_{avg}) per chain was calculated according to Equation 11 with o_{avg}: Average amount of blocks; M_n: Number average molecular weight determined *via* a GPC measurement; M_{CH2O}: Molecular weight of one oxymethylene units; and M_{EO}: Molecular weight of one oxyethylene unit.

$$o_{avg} = \frac{M_n - a_{monoCH2O}/100\times(M_{CH2O}+M_{EO})}{n_{CH2O}\times M_{CH2O}+n_{EO}\times M_{EO}} \quad \text{Eq. 11}$$

The relative number of mono-oxymethylene/mono-oxyethylene per block was determined according to Equation 12:

$$n_{mono} = \frac{a_{monoCH2O}}{o_{avg}} \quad \text{Eq. 11}$$

The range with a molecular weight above 900 g/mol was recalculated to the molecular weight of the corresponding copolymer fraction by referencing to polymer standards (polyoxyethylene diols) of known molecular weight (Figure S3). For copolymer **1,** a broad molecular weight distribution (PDI = 2.80) with a number average molecular weight (M_n) of 8,223 g/mol was found (Figure S4). Two low intensity signals were observed below 5,000 g/mol.

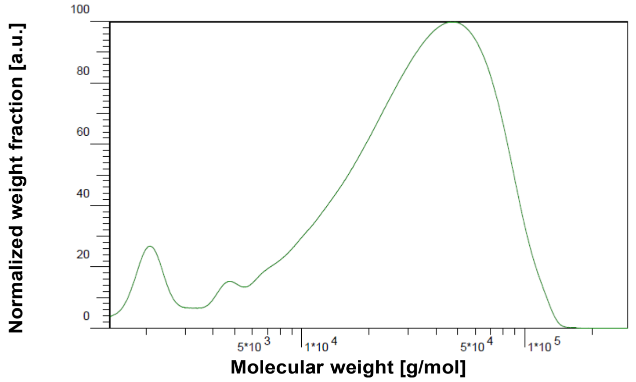

Figure S4 Normalised molecular weight distribution of copolymer **1** measured by GPC chromatography in chloroform (M_n = 8200 g/mol, PDI = 2.80).

Kinetics

To obtain insight into the reaction sequence, aliquots were withdrawn recurrently during the copolymerisation of polyoxyethylene diol **A** with trioxane and dioxolane to copolymer **1**. The aliquots were quenched through addition to an aqueous solution of triethylamine, and the composition of the mixtures was analysed by ^{1}H NMR spectroscopy. A plot of the concentrations of trioxane and dioxolane with time revealed a fast decrease (Figure S5). The consumption commenced immediately without induction period. Thus, the onset of the chain propagation must have been fast. Notably, a plot of the concentration of trioxane relative to dioxolane against time showed a constant value (Figure S6). This suggests

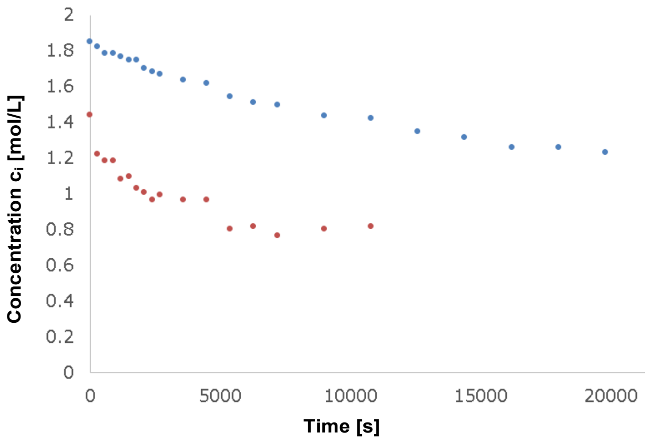

Figure S5. Time-concentration profile of trioxane (blue) and dioxolane (red) during the formation of copolymer **1**.

that trioxane and dioxolane were consumed in parallel. From this, we infer that the oxymethylene and oxyethylene moieties, which result from the ring opening of trioxane and dioxolane, were built into the growing polymer chain in a statistical manner. Please note that the ring opening of trioxane gives rise to formation of three oxymethylene moieties, and ring opening of dioxolane to formation of one oxymethylene and one oxyethylene moiety.

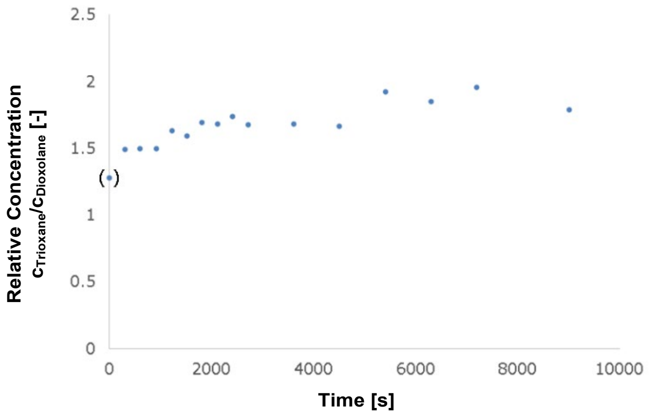

Figure S6. Ratio of the concentrations of trioxane and dioxolane during the formation of copolymer **1**. The decrease at t = 0 s is an artefact caused by evaporation of a small amount of dioxolane into the gas phase of the reactor.

Kinetic model

The condensation reactions were described with the following rate laws (Equation D1 to D9). To determine the rate constants of the initial rate of the PEG1000 consumption and the condensation reactions, the profile of the oligomer fractions was fitted with the kinetic model.

$$\frac{d[PEG1000]}{dt} = -2k_2[PEG1000][PEG1000] - k_2[PEG1000][Dimer] - k_2[PEG1000][Trimer] - k_2[PEG1000][Tetramer] - k_2[PEG1000][Pentamer] - k_2[PEG1000][Hexamer] - k_2[PEG1000][Heptamer] \quad \text{D1}$$

$$\frac{d[Dimer]}{dt} = +2k_2[PEG1000][PEG1000] - k_2[PEG1000][Dimer] - 2k_3[Dimer][Dimer] - k_3[Dimer][Trimer] - k_3[Dimer][Tetramer] - k_3[Dimer][Pentamer] - k_3[Dimer][Hexamer] \quad \text{D2}$$

$$\frac{d[Trimer]}{dt} = +k_2[PEG1000][Dimer] - k_2[PEG1000][Trimer] - k_3[Dimer][Trimer] - 2k_4[Trimer][Trimer] - k_4[Trimer][Tetramer] - k_4[Trimer][Pentamer] \quad \text{D3}$$

$$\frac{d[higher\ Oligomers]}{dt} = +\frac{d[Tetramer]}{dt} + \frac{d[Pentamer]}{dt} + \frac{d[Hexamer]}{dt} + \frac{d[Heptamer]}{dt} + \frac{d[Octamer]}{dt} \quad \text{D4}$$

$$\frac{d[Tetramer]}{dt} = +k_2[PEG1000][Trimer] + k_3[Dimer][Dimer] - k_2[PEG1000][Tetramer] - k_3[Dimer][Tetramer] - k_4[Trimer][Tetramer] - 2k_4[Tetramer][Tetramer] \quad \text{D5}$$

$$\frac{d[Pentamer]}{dt} = +k_2[PEG1000][Tetramer] + k_3[Dimer][Trimer] - k_2[PEG1000][Pentamer] - k_3[Dimer][Pentamer] - k_4[Trimer][Pentamer] \quad \text{D6}$$

$$\frac{d[Hexamer]}{dt} = +k_2[PEG1000][Pentamer] + k_3[Dimer][Tetramer] - k_2[PEG1000][Hexamer] - k_3[Dimer][Hexamer] \quad \text{D7}$$

$$\frac{d[Heptamer]}{dt} = +k_2[PEG1000][Hexamer] + k_3[Dimer][Pentamer] + k_4[Trimer][Tetramer] - k_2[PEG1000][Heptamer] \quad \text{D8}$$

$$\frac{d[Octamer]}{dt} = +k_2[PEG1000][Heptamer] + k_3[Dimer][Hexamer] + k_4[Trimer][Pentamer] + k_4[Tetramer][Tetramer] \quad \text{D9}$$

The concentration profiles of trioxane and dioxolane were described by the linearized rate law stated in Equation 14 (Eq. 14). For this purpose, the propagation reactions were translated into rate law (D10). The time-concentration profiles of trioxane and dioxolane were linearized according to Equation 13, and the rate constants and initial rates of the condensation reactions were determined.

$$\frac{d[Trioxane]}{dt} = -k_i[Trioxane][OH]^n \quad \text{D10a}$$

$$\frac{d[Dioxolane]}{dt} = -k_i[\mathrm{Dioxolane}][OH]^n \quad \text{D10b}$$

The rate of propagation reactions occurring for trioxane and dioxolane appeared to be independent of the concentration of hydroxyl groups. Hence, the reaction order in the concentration of hydroxyl groups was 0, so that the rate law is simplified to the differential Equation D11.

$$\frac{d[Trioxane]}{dt} = -k_i[\mathrm{Trioxane}] \quad \text{D11a}$$

$$\frac{d[Dioxolane]}{dt} = -k_i[\mathrm{Dioxolane}] \quad \text{D11b}$$

The profile of the trioxane and dioxolane concentrations is described by Equation 12 with $c_{t,i}$: Concentration of component *i* after a time of *t*; and $A_{i,ti}$: Integral recorded for component *i* after a time of *t*.

$$c_{i,t} = x_i A_{i,ti} \quad \text{Eq. 12}$$

This equation was transformed into Equation 13.

$$[Trioxane] = [\mathrm{Trioxane}]_0 e^{-k_i t} \quad \text{Eq. 13a}$$

$$[Dioxolane] = [\mathrm{Dioxolane}]_0 e^{-k_i t} \quad \text{Eq. 13b}$$

The latter was used to determine the rate constants of the propagation reactions from the slope of the linearized time-concentration profile.

$$ln\left(\frac{[Trioxane]}{[\mathrm{Trioxane}]_0}\right) = -k_i t \quad \text{Eq. 14a}$$

$$ln\left(\frac{[\mathrm{Dioxolane}]}{[\mathrm{Dioxolane}]_0}\right) = -k_i t \quad \text{Eq. 14b}$$

To determine the rate constants and the initial rates of the condensation reactions, the time-concentration profiles of trioxane and dioxolane were linearized according to Equation 13 and plotted in Figure S7.

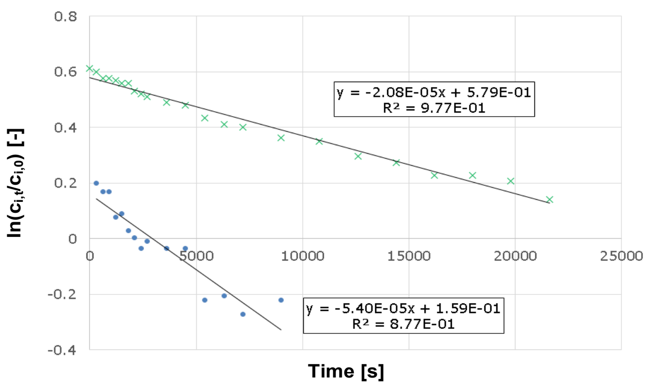

Figure S7 Linearized time-concentration profile of the trioxane (green) and dioxolane (blue) consumption during the formation of copolymer **1**.

Supplementary analytic data

Analytic data of copolymer 1

Yield: 93.617 g = 59 %
^{1}H-NMR ($CDCl_3$, 400.17MHz, 295K): δ = 4.92 – 4.71 (m, 2H), 3.85 – 3.79 (bs, 4H), 3.69 (s, 4H), 3.61 (s, 4H) ppm.
^{13}C-NMR ($CDCl_3$, 100,63MHz, 295K): δ = 66.92 – 67.57, 70.61, 92.10 – 92.78, 95.61 ppm.
Molecular weight (GPC): 8,223 g/mol
Degree of crystallinity (DSC): 32.46 %
Melting point (DSC): 41.06 °C

Analytic data for copolymer 2

Yield: 10.328 g = 34 %
^{1}H-NMR ($CDCl_3$, 400.17MHz, 295K): δ = 4.74 – 4.60 (m, 2H), 3.75 – 3.58 (bs, 4H), 3.56 (s, 4H), 3.51 (s, 4H) ppm.
^{13}C-NMR ($CDCl_3$, 100,63MHz, 295K): δ = 66.86 – 67.59, 70.63, 88.36 – 88.76, 92.12 – 92.49, 95.61 – 96.07 ppm.
Molecular weight (GPC): 1,767 g/mol
Decomposition temperature (TGA): 222.89 °C

Analytic data for copolymer 3

Yield: 10.328 g = 27 %
^{1}H-NMR ($CDCl_3$, 400.17MHz, 295K): δ = 4.87 – 4.66 (m, 2H), 3.70 – 3.69 (bs, 4H), 3.64 (s, 4H), 3.56 (s, 4H) ppm.
^{13}C-NMR ($CDCl_3$, 100,63MHz, 295K): δ = 66.83 – 67.61, 70.62, 88.33 – 89.15, 95.60 ppm.
Molecular weight (GPC): 2,239 g/mol
Decomposition temperature (TGA): 206.46 °C
Degree of crystallinity (DSC): 9.28 %
Melting point (DSC): 33.73 °C

Analytic data for copolymer 4

Yield: 26.492 g = 55 %
^{1}H-NMR ($CDCl_3$, 400.17MHz, 295K): δ = 4.88 – 4.71 (m, 2H), 3.77 – 3.75 (bs, 4H), 3.70 (s, 4H), 3.62 (s, 4H) ppm.
^{13}C-NMR ($CDCl_3$, 100,63MHz, 295K): δ = 66.85 – 67.64, 70.64, 88.34 – 89.16, 92.13 – 92.49, 95.62 ppm.
Molecular weight (GPC): 2,429 g/mol
Decomposition temperature (TGA): 203.13 °C
Degree of crystallinity (DSC): 9.91 %
Melting point (DSC): 43.13 °C

Analytic data for copolymer 5

Yield: 22.704 g = 32 %
^{1}H-NMR ($CDCl_3$, 400.17MHz, 295K): δ = 4.93 – 4.73 (m, 2H), 3.80 – 3.77 (bs, 4H), 3.71 (s, 4H), 3.63 (s, 4H) ppm.
^{13}C-NMR ($CDCl_3$, 100,63MHz, 295K): δ = 66.87 – 67.66, 70.65, 88.37 – 89.18, 92.14 – 92.51, 95.64 ppm.
Molecular weight (GPC): 2,652 g/mol
Decomposition temperature (TGA): 209.29 °C
Degree of crystallinity (DSC): 9.52 %
Melting point (DSC): 3.34 °C

Analytic data for copolymer 6

Yield: 26.505 g = 67 %
^{1}H-NMR ($CDCl_3$, 400.17MHz, 295K): δ = 4.77 – 4.62 (m, 2H), 3.77 – 3.66 (bs, 4H), 3.61 (s, 4H), 3.59 (s, 4H) ppm.
^{13}C-NMR ($CDCl_3$, 100,63MHz, 295K): δ = 66.81 – 67.55, 70.58, 88.30, 92.06 – 92.44, 95.56 ppm.
Molecular weight (GPC): 2,473 g/mol
Decomposition temperature (TGA): 209.36 °C
Degree of crystallinity (DSC): 15.72 %
Melting point (DSC): 10.99 °C

Analytic data for copolymer 7

Yield: 28.162 g = 59 %
^{1}H-NMR ($CDCl_3$, 400.17MHz, 295K): δ = 4.77 – 4.63 (m, 2H), 3.66 – 3.65 (bs, 4H), 3.61 (s, 4H), 3.53 (s, 4H) ppm.
^{13}C-NMR ($CDCl_3$, 100,63MHz, 295K): δ = 66.83 – 67.61, 70.60, 88.33 – 89.14, 92.11 – 92.47, 95.60 – 96.03 ppm.
Molecular weight (GPC): 3,300 g/mol
Decomposition temperature (TGA): 181.24 °C
Degree of crystallinity (DSC): 10.59 %
Melting point (DSC): 3.87 °C

Analytic data for copolymer 8

Yield: 39.979 g = 70 %
^{1}H-NMR ($CDCl_3$, 400.17MHz, 295K): δ = 4.91 – 4.72 (m, 2H), 3.79 – 3.76 (bs, 4H), 3.70 (s, 4H), 3.61 (s, 4H) ppm.
^{13}C-NMR ($CDCl_3$, 100,63MHz, 295K): δ = 66.85 – 67.64, 70.62, 88.35 – 89.16, 92.11 – 92.49, 95.62 ppm.
Molecular weight (GPC): 3,251 g/mol
Decomposition temperature (TGA): 174.21 °C
Degree of crystallinity (DSC): 7.88 %
Melting point (DSC): 1.33 °C

Analytic data for copolymer 9

Yield: 36.947 g = 46 %
^{1}H-NMR ($CDCl_3$, 400.17MHz, 295K): δ = 4.90 – 4.70 (m, 2H), 3.77 – 3.74 (bs, 4H), 3.68 (s, 4H), 3.60 (s, 4H) ppm.
^{13}C-NMR ($CDCl_3$, 100,63MHz, 295K): = δ = 66.76 – 67.54, 70.36, 76.79 – 77.42, 88.66 – 89.07, 92.03 – 92.39, 95.52 ppm.
Molecular weight (GPC): 3,512 g/mol
Decomposition temperature (TGA): 170.55 °C
Degree of crystallinity (DSC): 8.48 %
Melting point (DSC): 2.87 °C

Analytic data for copolymer 10

Yield: 24.353 g = 57 %
^{1}H-NMR ($CDCl_3$, 400.17MHz, 295K): δ = 4.89 – 4.71 (m, 2H), 3.80 – 3.76 (bs, 4H), 3.69 (s, 4H), 3.62 (s, 4H) ppm.
^{13}C-NMR ($CDCl_3$, 100,63MHz, 295K): δ = 66.83 – 67.54, 70.59, 92.07, 95.58 – 96.01 ppm.
Molecular weight (GPC): 3,907 g/mol
Decomposition temperature (TGA): 211.17°C
Degree of crystallinity (DSC): 36.84 %
Melting point (DSC): 42.11 °C

Analytic data for copolymer 11

Yield: 28.449 g = 60 %
^{1}H-NMR ($CDCl_3$, 400.17MHz, 295K): δ = 4.89 – 4.71 (m, 2H), 3.80 – 3.76 (bs, 4H), 3.69 (s, 4H), 3.62 (s, 4H) ppm.
^{13}C-NMR ($CDCl_3$, 100,63MHz, 295K): δ = 66.76 – 67.50, 70.54, 88.25 – 89.07, 92.02 – 92.40, 95.53 ppm.
Molecular weight (GPC): 3,976 g/mol
Decomposition temperature (TGA): 200.03 °C

Degree of crystallinity (DSC): 32.12 %
Melting point (DSC): 40.34 °C

Analytic data for copolymer 12

Yield: 37.966 g = 69 %
^{1}H-NMR ($CDCl_3$, 400.17MHz, 295K): δ = 4.80 – 4.67 (m, 2H), 3.70 – 3.65 (bs, 4H), 3.57 (s, 4H) ppm.
^{13}C-NMR ($CDCl_3$, 100.63MHz, 295K): δ = 66.68, 67.33 – 67.41, 70.46, 88.16 – 88.98, 91.92 – 92.30 ppm.
Molecular weight (GPC): 4,642 g/mol
Decomposition temperature (TGA): 197.69°C
Degree of crystallinity (DSC): 23.26 %
Melting point (DSC): 35.98 °C

Analytic data for copolymer 13

Yield: 45.143 g = 66%
^{1}H-NMR ($CDCl_3$, 400.17 MHz, 295 K): δ = 4.85 – 4.71 (m, 2H), 3.68 – 3.67 (bs, 4H), 3.64 (s, 4H) ppm.
^{13}C-NMR ($CDCl_3$, 100,63MHz, 295K): δ = 66.92 – 67.57, 70.70, 88.75 – 89. 15, 92.13 – 92.48, 95.57 ppm.
Molecular weight (GPC): 4,549 g/mol
Decomposition temperature (TGA): 201.17 °C
Degree of crystallinity (DSC): 22.94 %
Melting point (DSC): 37.55 °C

Table S2 Multi-block POM-PEG copolymers synthesised from polyoxyethylene diols, trioxane, and dioxolane. Sulphur trioxide coordinated by dimethyl formamide was used to initiate the copolymerisation.

Copolymer	Eq_{Tx}[a] [-]	PEG [g/mol]	M_n[b] [g/mol]	x_{EO}[c] [mol-%]	$x_{monoCH2O}$[d] [mol-%]	x_{CH2O}[e] [mol-%]	n_{EO}[f] [-]	n_{CH2O}[g] [-]	n_{mono}[h] [-]	o_{avg}[i] [-]	Yield [w-%]
**1**	4.9	1,078	8,223	87.4	2.3	20.3	27.2	7.5	0.8	3.4	59
**10**	1.1	1,078	3,907	90.9	5.6	3.5	58.4	2.4	3.9	1.3	57
**11**	3.0	1,078	3,976	83.2	3.2	13.5	31.8	5.5	1.3	2.4	60
**12**	4.8	1,078	4,642	76.9	2.4	20.7	28.3	8.0	0.9	3.0	69
**13**	9.8	1,078	4,549	87.5	2.3	10.2	27.2	7.5	0.8	3.1	66
**6**	1.0	364	2,473	74.2	12.3	13.5	18.4	4.1	3.7	2.1	67
**7**	3.1	364	3,300	58.7	6.6	34.7	10.0	6.8	1.3	4.5	59
**8**	5.0	364	3,251	58.6	4.9	36.5	11.3	7.9	1.0	4.0	70
**9**	10.1	364	3,512	58.2	5.1	36.8	11.0	7.8	1.1	4.4	46
**2**	1.0	136	1,767	61.7	18.6	19.7	9.6	4.5	4.2	2.0	34
**3**	3.0	136	2,239	46.1	8.3	45.6	5.5	7.0	1.2	4.1	27
**4**	4.9	136	2,429	41.7	6.3	52.0	5.1	7.9	0.9	4.6	55
**5**	10.1	136	2,652	44.0	6.9	49.0	5.3	7.3	1.0	5.0	32

a: Equivalents of trioxane, b: Number average molecular weight, c: Molar amount of oxyethylene units incorporated, d: Molar fraction of mono-oxymethylene units incorporated, e: Molar fraction of oxymethylene units incorporated, f: Length of oxyethylene blocks, g: Length of oxymethylene blocks, h: Average number of mono-oxymethylene units per block, i: Average number of repeating blocks per copolymer chain.

Chain elongation

Copolymer **1** was chain extended with toluene diisocyanate in the presence of DBTL as catalyst. Full conversion of isocyanate groups was confirmed with *in-situ* IR spectroscopy. For this, TDI was added in four pulses to a solution of copolymer **1** in dichlorobenzene, thereby following the consumption of isocyanate with *in situ* IR spectroscopy (Figure S8). The consumption of isocyanate was followed by evaluating the intensity of the IR-bands between 2280 and 2250 cm^{-1} assigned to isocyanate. TDI was added in four pulses over the course of 3.5 hours. Thereafter the reaction mixture was left stirring at 80 °C for another 14.5 hours. Polyurethane **14** was then precipitated from petrol ether. Characterization by ^{1}H-NMR spectroscopy revealed that polyurethane **14** was composed of oxymethylene, oxyethylene, and polyurethane moieties. The molecular weight distribution of polyurethane **14** was determined with gel permeation chromatography (GPC) to be M_n, 10.6 x 10^3 g/mol.

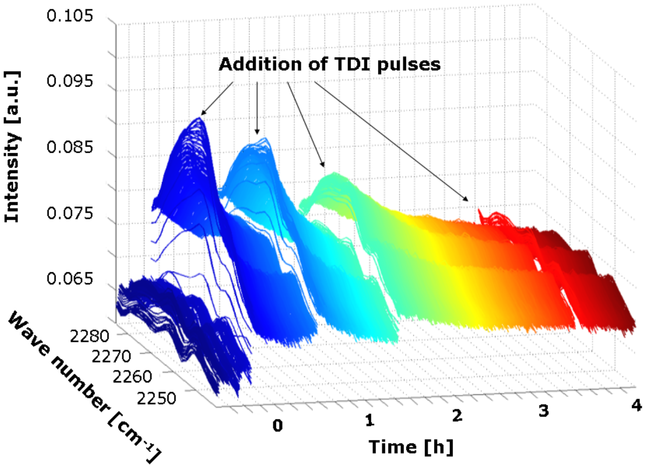

Figure S8. Isocyanate region of the time-resolved *in situ* IR spectra during the pulse-wise addition of TDI to copolymer **1**.
